# New clinical trial design in precision medicine: discovery, development and direction

**DOI:** 10.1038/s41392-024-01760-0

**Published:** 2024-03-04

**Authors:** Xiao-Peng Duan, Bao-Dong Qin, Xiao-Dong Jiao, Ke Liu, Zhan Wang, Yuan-Sheng Zang

**Affiliations:** Department of Medical Oncology, Changzheng Hospital, Naval Medical University, Shanghai, China

**Keywords:** Drug development, Diagnostics, Outcomes research, Molecular medicine, Cancer genomics

## Abstract

In the era of precision medicine, it has been increasingly recognized that individuals with a certain disease are complex and different from each other. Due to the underestimation of the significant heterogeneity across participants in traditional “one-size-fits-all” trials, patient-centered trials that could provide optimal therapy customization to individuals with specific biomarkers were developed including the basket, umbrella, and platform trial designs under the master protocol framework. In recent years, the successive FDA approval of indications based on biomarker-guided master protocol designs has demonstrated that these new clinical trials are ushering in tremendous opportunities. Despite the rapid increase in the number of basket, umbrella, and platform trials, the current clinical and research understanding of these new trial designs, as compared with traditional trial designs, remains limited. The majority of the research focuses on methodologies, and there is a lack of in-depth insight concerning the underlying biological logic of these new clinical trial designs. Therefore, we provide this comprehensive review of the discovery and development of basket, umbrella, and platform trials and their underlying logic from the perspective of precision medicine. Meanwhile, we discuss future directions on the potential development of these new clinical design in view of the “Precision Pro”, “Dynamic Precision”, and “Intelligent Precision”. This review would assist trial-related researchers to enhance the innovation and feasibility of clinical trial designs by expounding the underlying logic, which be essential to accelerate the progression of precision medicine.

## Introduction

In 2003, the human genome project (HGP) was completed, leading to a deeper understanding of clinical medicine. The accomplishment of HGP has been considered as the cradle of precision medicine.^1^ In 2011, the National Research Council of the United States proposed the concept of “precision medicine” in the article “Toward Precision Medicine: Building a Knowledge Network for Biomedical Research and a New Taxonomy of Human Disease”. In 2015, Barack Obama launched the precision medicine initiative as a bold new research effort to revolutionize health and disease treatment. This program promoted the rapid dissemination of precision medicine worldwide.^2^ Moreover, the availability of high-throughput gene sequencing technology,^3^ as well as the importance of proteomics, metabolomics, transcriptomics, and epigenetics spurred interest in thoroughly understanding human disease,^4–8^ eventually accelerating the development of precision medicine. Precision medicine has been defined in a variety of ways depending on the perspective of researchers. Commonly, precision medicine is defined as an evolving approach to disease prevention and treatment that incorporates an individual’s genetic, environmental, and lifestyle factors.^9^ This strategy yields useful information that moves from the conventional “one-size-fits-all” approaches to selective approaches governed by individual variability.^10^ This novel healthcare model has the capacity to facilitate the efficient and accurate identification of the optimal care for individual patients. Although the definition has evolved over several years, genomics information often serves as the basis of precision medicine and is used to develop individualized precision management, especially for precision treatment.^11^

Traditionally, clinical treatment strategies have been approved based on average-population-benefit decisions derived from the randomized clinical trials of unselected patients, which were the cornerstone of traditional drug approvals. Tissue-of-origin trials are drug-centered, which refers to investigations that provide one drug to all patients. Patients are selected for trial inclusion based on commonalities such as disease. However, as multi-omics sequencing technology has developed and become widely used, it has been increasingly recognized that individuals with certain diseases are complex and different from each other.^12,13^ Due to the significant heterogeneity of participants enrolled in traditional “one-size-fits-all” trials, patient-centered trials that could provide optimal therapy customization to individuals with specific biomarkers were developed. With increased interest and effort being put toward patient-centered trials, it is essential to recognize the importance of genomic alterations and further develop biomarker-guided therapies in clinical trials.^14^ Significant methodological advances in biomarker-guided clinical trial designs have been made toward patient-centered trials, including the basket, umbrella, and platform trial designs under the master protocol framework.^15,16^ A master protocol refers to a single, overarching design that can assess multiple hypotheses with the general goal of improving efficiency and constructing uniformity through standardized trial procedures during the development and evaluation of different interventions.^17^ Master protocols are often divided into three new trial designs: basket, umbrella, and platform trials. A basket trial refers to using the same drug or intervention to treat patients who share a common characteristic, such as a genetic alteration or a specific biomarker.^18^ Currently, basket trials are commonly used in the field of precision oncology, and they have been formulated to investigate the efficacy of molecular-targeted therapies for oncogene-defined subsets of cancers across different tumor histologies.^19–21^ An umbrella trial refers to designs where multiple therapies or interventions for patients with a certain disease are stratified into subgroups according to different characteristics that include clinical features and molecular alternations.^15,20^ In 2018, the Food and Drug Administration (FDA) released a guidance document describing recommendations for basket and umbrella trials, providing support for these new designs. A recent investigation found that the number of basket and umbrella trials has rapidly increased, suggesting a wider dissemination of these trial designs.^22^ Both basket and umbrella trials use a molecular screening protocol that either permits the enrollment of different diseases with a certain characteristic or a certain disease with different subtypes. However, both of these trials were designed using a fixed protocol at a specific time point. This fixed model greatly limits the efficiency of clinical trials with the rapid development of precision medicine, requiring a new clinical trial design that would be adaptable and responsive to emerging evidence. Hence, a new trial design called the platform trial has recently been proposed, which could be used to greatly accelerate the efficiency of clinical trials. Platform trials, also referred to as multi-arm, multi-stage design trials, are trials that continuously assess several interventions against a certain disease and adapt the trial design based on the accumulated data.^23,24^ This design allows for the early termination of ineffective interventions and flexibility in adding new interventions during the trial.

Despite the rapid increase in the number of basket, umbrella, and platform trials, the current clinical and research understanding of these new trial designs, as compared with traditional trial designs, remains limited. The majority of the research has focused on methodologies, but there is a lack of in-depth insight concerning the underlying biological logic of these new clinical trial designs. Therefore, we provide this comprehensive review of the discovery and development of basket, umbrella, and platform trials and their underlying logic from the perspective of precision medicine. We then discuss the future directions of these new trial designs in view of the “precision pro”, “dynamic precision”, and “intelligent precision”. By expounding the underlying logic, this review aims to assist trial-related researchers to enhance the innovation and feasibility of clinical trial designs. This review will also support cancer research-related scientists in understanding the logic of clinicians, thereby improving the transformation efficiency.

## Discovery: clinical dilemma prompting an exploration of new biomarker-guided trial design

**Fig. 1:**
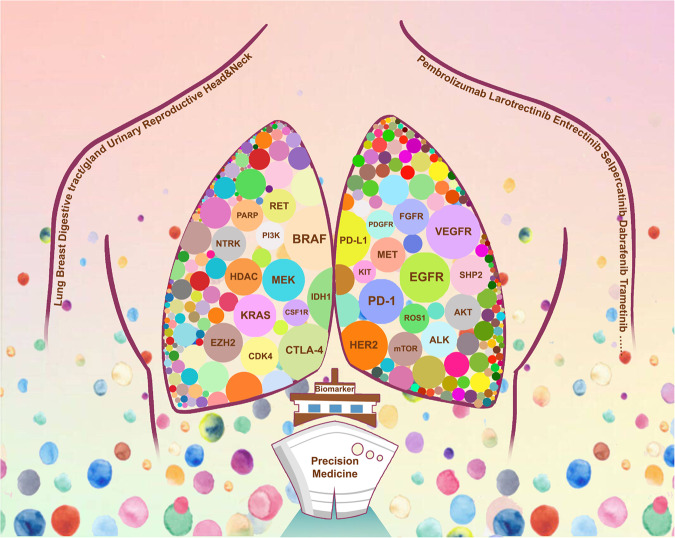
The biological logic of new biomarker-guided clinical trial design in precision medicine. The essence of precision medicine is to explore the unknown relationship between drugs, targets, and diseases in the human body. The left and right arms represent the drugs and diseases, respectively. The circles distributed throughout the lungs and body represent the therapeutic targets. The world of precision medicine in human body is an undeveloped ocean, containing extensive therapeutic targets represented by colorful circles based on next-generation sequencing and other biological technologies. The diseases such as lung cancer are classified into different subtypes based on different therapeutic targets. The molecular subtype-guided therapy in a certain disease is the biological logic of umbrella trial design. A certain target may appear in the lungs and other parts of the body with similar biological characteristics. Based on the successful treatment experience of lung cancer, exploring the therapeutic potential of a certain target for the disease of other body parts is the underlying logic of the basket trial design. The dynamic concept conveyed by the diffuse distribution of circles in the ocean is the core of platform trial design. The biomarker-guided ship of precision medicine clinical trial design is constantly advancing, riding the wind and waves in the undeveloped ocean of precision medicine

The current landscape of precision medicine was established based on the understanding of potential molecular phenotypes in diseases and attempts to target these molecular phenotypes (Fig. 1). The development of precision medicine was driven by the mapping of the human genome and the maturity of next-generation sequencing (NGS).^25,26^ Advancements in sequencing technologies have significantly improved the ability to rapidly and comprehensively identify genetic phenotypes. In particular, recent high-throughput next-generation sequencing advancements have promoted the rapid and simultaneous detection of all types of gene alterations, mainly including gene mutations, rearrangements, and copy number changes.^27,28^ This has fueled more efforts towards precision medicine, in which therapies are chosen in accordance with genetic alterations. These innovative treatments are commonly referred to as biomarker-guided therapies, and an increasing number of diseases may derive clinical benefits from this strategy. For example, a prospective clinical sequencing project of 10,000 patients led by the Memorial Sloan-Kettering Cancer Center (MSKCC) showed that there are potentially treatable genetic changes in over 36% of patients with advanced cancers.^29^ In addition, innovations in the development of drugs that target specific disease-driving gene alterations have accelerated the introduction and expansion of biomarker-guided therapies. Historically, this treatment strategy originated in oncology and has evolved and matured in the field of precision oncology. It is now also applied in multiple other clinical scenarios, such as diabetes, cardiovascular, kidney, and neurological diseases.^30–33^

The proof-of-concept for biomarker-guided therapy was initiated from the success of imatinib for patients with chronic myelogenous leukemia (CML) harboring the BCR-ABL translocation. This genomic-driven targeted therapy resulted in a remarkable survival improvement, leading the life expectancy of patients with CML to approach that of the general population.^34^ Subsequently, drugs targeting the EGFR, ALK, ROS1, MET-mutant lung cancer, HER2-overexpression breast cancer and gastric cancer, and BRAF V600E mutant melanoma have dramatically improved the prognosis of these patients. These significant clinical benefits from therapies that target patient genomic aberrations have propelled a paradigm of choosing therapy strategies based on an individual’s molecular profile. Subsequent clinical trials have begun to enroll patients based on their genetic phenotypes, and many standardized biomarker-guided treatment protocols have been developed from these clinical trials. For instance, evidence from several large-scale clinical trials has promoted first/second/third EGFR-tyrosine kinase inhibitor (TKI) as the standard-of-care among non-small-cell lung cancer (NSCLC) patients with EGFR-sensitive mutation.^35–37^

**Fig. 2:**
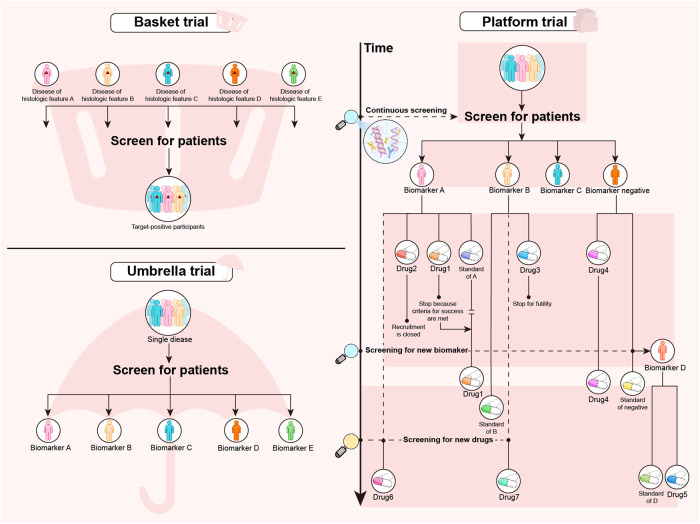
The pattern of the basket, umbrella, and platform trial design. The basket trials aim to evaluate the efficacy of a certain biomarker in multiple diseases or different tissue types (such as cancer). Recruitment is completed by screening patients with the same therapeutic target through next-generation sequence and other biologic technologies. The umbrella trials aim to reclassify a certain traditional disease type based on potential therapeutic biomarkers. Recruited patients are assigned to different molecular subgroups and matched with corresponding drugs. The platform trials aim to continuously screen biomarkers and drugs for a certain disease. The biomarker subgroups and treatment types are dynamically added or removed under the constraints of the master protocol. At the initial stage, screening for biomarkers such as A, B, C, and negative, is conducted and patients are assigned to the biomarker-guided cohorts. Considering that each target may correspond to multiple drugs, a control group and one to multiple experimental groups (drug 1 and 2 in biomarker A group, drug 3 in biomarker B group, drug 4 in biomarker negative group) are set up in each cohort. If the experimental drug 2 is more effective than the current standard therapy of biomarker A group, it will replace the original standard as the new control group. During the course of trials, new treatment cohorts can be included in the corresponding biomarker-guided group if new drug 5 or 6 are available. When new biomarker D and corresponding drug 7 are available, the new biomarker D-guided group will be established. If a biomarker-guided group currently lacks standard control treatment, a single experimental group with efficacy termination threshold set is considered feasible

However, some standard regimens, even under the guidance of phase III clinical trials, have fallen far behind the growing therapeutic demand. The difficulties associated with the approvals of new drugs, as well as the long duration of these processes, also exacerbate the dilemma.^38,39^ In addition, conventional trial designs cannot be used to assess the efficacy of one regimen across different diseases or that of multiple regimens in a certain disease but with different features. Therefore, the efficient exploration of new trial designs on the therapeutic potential of drugs is a concern for trial-related clinicians and researchers. Master protocol frameworks have been proposed as a vital strategy to comprehensively and adaptively evaluate treatments in precision medicine.^15,16^ A typical representative model of a master protocol has emerged that includes basket, umbrella, and platform trials (Fig. 2).^15,16^ Recently, the number of these new trial designs has increased dramatically, and it is assumed that this trend will persist in the following years.^22^

### Basket trial design guided by the pan-cancer proliferation-driven molecular phenotype

In oncology research, therapies based on similar molecular alterations distributed in different anatomical cancer types accelerate the clinical expansion of antitumor drugs, such as the approval indications of imatinib across multiple cancer types and the progress of the molecular analysis for therapy choice (MATCH) plan that matches drugs with molecular phenotypes.^40–43^ Specifically, in 2014, the American Association for Cancer Research (AACR) proposed the “basket trial”, a phase II clinical trial that classifies treatments according to the universal and proliferation-driven molecular phenotype rather than pathology. The principle of a basket trial design is derived from a deep understanding of the pan-cancer proliferation-driven molecular phenotype.

The overexpression of HER2 in breast and bladder cancers is associated with chemotherapy resistance, elucidating the pan-cancer proliferation-driven molecular phenotype. In 1987, Di Fiore et al. discovered the proto-oncogenic effect of HER2 protein.^44^ The amplification or overexpression of HER2 was identified in 20–30% of patients with breast cancer and was associated with a lower chemotherapy remission rate and duration than those in patients with HER2-negative disease.^45–47^ Fortunately, anti-HER2 therapy combined with chemotherapy has been shown to prolong the duration of response and overall survival of breast cancer patients with HER2 overexpression for three and five months, respectively.^48^ Similarly, the rate of strong HER2 membrane staining (IHC2 + /3 + ) in advanced bladder cancer has been reported to be ~26%, which is similar to that in metastatic breast cancer, and chemotherapy resistance has been reported.^49^ The addition of trastuzumab to chemotherapy has been shown to significantly reduce the tumor size of bladder cancer with HER2 overexpression.^50^ Faced with the same biological characteristics and therapeutic prognosis among different tumor species, researchers have become aware of the cross-tumor proliferation-driven capability of this particular molecular phenotype, and they have begun to explore the therapeutic value of HER2 overexpression in pan-cancer. Inevitably, the amplification or overexpression rates of HER2 in ovarian, endometrial, pancreatic, colon, gastric, small cell lung, renal, and prostate cancers ranges from ~10–40%, and the standardized evaluation result of pan-cancer is similar.^51–59^ Furthermore, anti-HER2 combined with chemotherapy has been reported to achieve objective response rates (ORR) of 7.3%, 24.5%, and 47% in patients with HER2 overexpression in ovarian cancer, NSCLC, and gastric and gastroesophageal junction cancers, respectively.^58,60–62^ The “single-target to multi-drugs” model, which extended the benefits of chemotherapy-refractory HER2 overexpression in breast and bladder cancers to pan-cancer, was the prototype for the basket trial.

The comparison between the limited efficacy of chemotherapy, non-ALK-TKI therapy, and the high effectiveness of ALK-TKI therapy indicated the dominant proliferation-driven position of ALK fusion mutations in lung cancer, which, in basket trials, was the key condition for using ALK inhibitors alone without chemotherapy. The ALK mutation was initially identified in anaplastic large-cell lymphoma and was named “ALK lymphoma” based on the morphological homogeneity.^63,64^ However, because of the preferred chemotherapy response of lymphoma and a lack of awareness of the proliferation-driven molecular phenotype, the therapeutic value of the ALK fusion mutation remained unknown until the ALK-EML4 fusion was distinguished in NSCLC in 2007.^65^ The limited remission rates of conventional therapies indicate that ALK fusion mutations have powerful proliferation-driven capacities beyond growth factors and cell division signals.^66,67^ Indeed, this was confirmed by the excellent 57% ORR following treatment of ALK fusion NSCLC with ALK inhibitors.^68^ In 2012, the concept of “ALKoma” to define solid tumors with ALK mutations was proposed, where the ALK fusion mutation was recognized as a pan-cancer therapy goal due to its strong proliferation-driven ability. The ALK inhibitor, crizotinib, achieved 90% and 86% ORR and 80% and 36% complete response in anaplastic large-cell lymphoma (ALCL) and inflammatory myofibroblastoma (IMT), respectively.^69^ Subsequently, ALK inhibitors were shown to be effective in malignant peritoneal mesothelioma, neuroblastoma, renal cell carcinoma, colorectal cancer, and melanoma.^70–72^ The high remission rate of ALK inhibitors for ALK-driven NSCLC, ALCL, and IMT unlocked the “single-target to single-drug” model in the epoch dominated by chemotherapy, which was the most typical pattern of basket trials.

Considering the clinical bottleneck of poor response to conventional treatment as an opportunity, the basket trial identified the qualitative and quantitative proliferation-driven ability of the molecular phenotype in different tumor species and extended its application to pan-cancer. In contrast to traditional targeted therapy that focuses on the targets in one specific disease, basket trials pay more attention to the commonalities of targets in pan-cancer. The off-label attempt of basket trials brings hope to patients faced with the treatment dilemma. In addition, basket trials may provide initial proof-of-principle evidence for the clinical treatment potential of newly discovered disease-driven targets, especially for uncommon or orphan gene alternations.^73^ Moreover, the emergence of basket trials has made it possible to conduct drug development for low-frequency gene alternations that have been previously unexamined. For example, in 2018, a basket trial first demonstrated that larotrectinib had a remarkable and durable antitumor activity among patients with tropomyosin receptor kinase (TRK) fusion-positive cancer, regardless of the histology and age.^74^ Furthermore, a basket trial also allows for the initial screening of potential efficacy of a regimen to target specific alterations across multiple tumor types in order to guide subsequent disease-specific traditional trials. In a basket trial for vemerafinib in BRAF V600E-mutated pan-cancer, the results showed that vemerafinib showed activity in NSCLC and other histologies, but not in colorectal cancer.^75^ A follow-up conventional trial was then conducted separately in an NSCLC cohort with a large sample size.^76^ Finally, a basket trial can yield vital data to support a new standard regimen for rare cancers with specific targets. A basket trial reported in 2015 was the first to elucidate the efficacy of anti-BRAF therapy in patients with Erdheim-Chester disease and Langerhans cell histiocytosis and BRAF V600E.^75^ Previously, these two types of cancers lacked a standard recommendation, whereas this basket trial established a standard regimen for these diseases. Depending on a high enrollment efficiency across tumor anatomical species, the basket trial has been recognized by the FDA as an effective pathway for approving rare antitumor therapies.^77^ In the past 6 years, pan-cancer indications for pembrolizumab, larotrectinib, entrectinib, selpercatinib, dorstarlimab-gxly, envafolimab, serplulimab, dabrafenib, and trametinib have been approved in succession,^72,74,78–88^ indicating that basket trials are ushering in great opportunities.

Although basket trials are less common in nononcology fields, the concept of basket trials has also been applied to other nononcology diseases, including Alzheimer’s disease,^89,90^ vasculitides,^91^ metabolic diseases,^92,93^ and infectious diseases.^94,95^ For example, basket trials have been used in the assessment of the effectiveness of interventions that focus on specific pathophysiological mechanisms in Alzheimer’s disease. It should be emphasized that the utilization of basket trials in the nononcology field is currently constrained, and additional investigation is required to investigate the particular advantages and challenges that pertain to these fields.

### Umbrella trial design guided by molecular phenotypes of a certain disease

Due to shared genetic alterations across different cancer types, the basket trial design was developed with the core theme of “treating different diseases with the same treatment”. In contrast, the umbrella trial design was developed with the core theme of “treating the same disease with different treatments” due to the different molecular phenotypes of a certain disease.^16^ Thus, the umbrella trial was designed to evaluate multiple interventions within a particular disease in a single trial. The principle of the umbrella trial design stems from a deep understanding of disease heterogeneity, including genomic heterogeneity and clinical phenotypic diversity.^15,20^ For example, lung cancer was initially treated as a whole, but with varying outcomes. The treatment outcomes of lung cancer were then significantly improved by using different treatment approaches when lung cancers were categorized into different subtypes that included adenocarcinoma, squamous cell carcinoma, and small cell lung cancer. In the era of precision medicine, various gene mutations associated with lung adenocarcinoma, such as EGFR, ALK, MET, and ROS1, have been observed, and remarkable efficacy improvements by administering targeted therapies based on specific gene mutations have been achieved.^96,97^ Previously, a single traditional trial targeting a specific genetic phenotype or clinical characteristic was conducted, which was time-consuming and hindered the rapid clinical application of effective drugs or interventions. Umbrella trials have effectively addressed this issue.

Although the AACR formally proposed the “umbrella trial” concept in 2014, this trial design had been employed for a long time prior to this event. In 2006, the biomarker-integrated approaches of targeted therapy for lung cancer elimination (BATTLE) trial was initiated, which was a landmark umbrella trial in the field of precision oncology. The BATTLE trial was designed to evaluate multiple targeted therapies simultaneously in patients with NSCLC based on individual specific molecular profiles.^98^ All patients with NSCLC were assigned into four subgroups: KRAS/BRAF mutation, VEGF/VEGFR2 overexpression, RXR/CyclinD1 overexpression/CCND1 amplification, as well as EGFR alteration) to test the efficacy of the specific targeted therapy. BATTLE was the first umbrella trial to identify which treatments were most effective for specific genetic subgroups using a biomarker-driven approach. The successful implementation of early umbrella trials showcased the efficiency of this trial design in the era of precision medicine, subsequently leading to the initiation of numerous similar design trials.^99–102^ These trials showed the potential of umbrella trial designs to enhance the efficiency and effectiveness of clinical trials in the pursuit of precision medicine and to improve cancer treatments.

With advancements in precision medicine, there was a growing recognition that a one-size-fits-all strategy may not be suitable for all patients with a certain disease. The umbrella trial is a model that embodies the concepts of precision medicine and epitomizes the efficient implementation of precision medicine.^20,103,104^ Umbrella trials allow for the assessment of personalized treatment strategies by considering the specific characteristics or biomarkers of each patient. Additionally, as a valuable trial design, the umbrella trial design also addresses several issues in conventional trials. First, the diversity of diseases, also called heterogeneity, is under consideration in umbrella trials. An increasing number of diseases have been shown with significant heterogeneity, including different disease subtypes and molecular phenotypes. Typically, patients with a certain disease are enrolled into the arm that is most appropriate for their specific characteristics under a prespecified treatment arm design. Each arm is evaluated separately, and the trial may have a hierarchical statistical analysis plan to compare the effectiveness of different interventions against a common control group or standard-of-care. Second, the efficiency and resource optimization of clinical trials in precision medicine are improved. Umbrella trials provide a highly efficient method to evaluate multiple interventions simultaneously in a single trial. By incorporating multiple treatment options, investigators can collect comparative efficacy data rapidly and without the requirement for separate trials for each intervention. In addition, in traditional trials, it is expensive and time-consuming to perform individual trials for each intervention. In contrast, umbrella trials allow for the shared utilization of infrastructure, resources, and patient populations, resulting in cost and time efficiencies. Moreover, new effective biomarker-guided therapy regimens can be discovered using umbrella trials for one specific disease and can then be expanded to other types of diseases using basket trials, thus achieving the maximization and optimization of precision medicine.

In addition to basket or umbrella trial designs alone, the exploration of the combination of basket trials with umbrella trials has been an ongoing area of interest in precision medicine. The pooled objective of integrating these two trial designs is to develop a more comprehensive and personalized approach to select the optimal treatment. By incorporating the basket trial concept within an umbrella trial framework, investigators can assess multiple regimens simultaneously across different tumor types and genetic alterations. The MATCH conducted by the National Cancer Institute (NCI) was a notable trial design that integrated basket and umbrella trial designs; patients with refractory cancer were assigned to different subgroups according to the specific molecular alteration.^105,106^ Since 2015, NCI-MATCH has been in progress, with the primary objective of assessing a tumor-agnostic approach in the selection of treatments based on genetic alteration. To date, there are nearly 37 molecular subgroups in the NCI-MATCH trial, which is considered the largest umbrella trial worldwide. In 2022, FDA grants accelerated the approval of dabrafenib in combination with trametinib for unresectable or metastatic solid tumors with the BRAF V600E mutation, which was supported by data from the NCI-MATCH trial.^86^ Nevertheless, it is crucial to note that this combination design is still the subject of ongoing trials led by governmental institutions, and further studies are required to fully understand and maximize its value in precision medicine.

### Platform trial design guided by the dynamic perspective of precision medicine

Both basket and umbrella trials are revolutionary innovations for the accelerated development of precision therapeutic drugs and the advancement of precision medicine. Basket and umbrella trials have significantly accelerated drug development or drug indication approvals, but they rely on drugs (or interventions) that are limited to a certain time point, resulting in lack of dynamic adaptation to the latest evidence. In the era of precision medicine, the importance of a dynamic perspective is increasingly emphasized in view of disease evolution and the rapid emergence of novel drugs (or interventions), thus giving rise to new clinical trial designs called platform trials. A platform trial is a flexible and adaptive clinical trial design that allows for the simultaneous evaluation of multiple interventions or treatment strategies against a single control arm for a specific disease within a unified framework.^23,24^ Within the prespecified protocol, it is possible to add interventions that show promise or remove ones with insufficient evidence of activity over time, enabling ongoing evaluation and optimization based on innovative and emerging scientific knowledge or advancements in treatment strategies.^107^

The primary advantages of platform trials are flexibility, adaptability, and the capacity to dynamically adjust trial designs based on accumulating evidence throughout the trial. For example, the STAMPEDE trial, initiated in 2005, was the first multi-arm platform trial conducted in high-risk localized or metastatic prostate cancer.^108^ The last patient for the STAMPEDE trial was recruited on March 31, 2023, marking the official completion of the largest platform trial, which enrolled nearly 12,000 participants and lasted for 18 years. A notable feature of the STAMPEDE trial was its adaptive design. Although only six arms were included in STAMPEDE when it was initiated in 2005, nearly 11 interventions had been investigated when the trial was closed in 2023. This trial generated significant research findings that resulted in treatment-changing guidelines for patients with advanced prostate cancer. For instance, the STAMPEDE trial demonstrated the benefits of adding docetaxel chemotherapy, abiraterone acetate, and androgen deprivation therapy (ADT) to the standard-of-care for improving overall survival, all of which were then cited into the guidelines for prostate cancer as the recommended stand-of-care.^109–111^ Moreover, platform trials often incorporate a biomarker-guided approach in which patients are stratified according to specific molecular genotype or genetic alteration. This feature enables the evaluation of interventions in selected patient populations, eventually leading to personalized treatment approaches. For example, the GBM-AGILE is a novel multi-arm platform trial designed to evaluate a therapy based on biomarker status, including the EGFR alteration and the MGMT promoter methylation status, among patients with glioblastoma.^112^

A platform trial design exhibits several advantages for clinical investigation in precision medicine.^113–115^ First, similar to umbrella trial design, the platform trial design offers clinical investigation efficiency by assessing multiple interventions simultaneously within a single trial. This design accelerates the research process as well as reduces the duplication of efforts, eventually optimizing resource utilization. Second, platform trials facilitate rapid learning and informed decision-making by continuously monitoring and analyzing interim data of the trial and external emerging evidence. This allows for the timely adjustment of treatment strategies, interventions, and trial processes. Third, platform trials offer improvements in statistical power. Platform trials require larger sample sizes because they evaluate multiple interventions, which can enhance the accuracy for estimating treatment effects and enable the detection of smaller but clinically significant differences. Finally, platform trials promote collaboration and data sharing among researchers and clinicians. For example, 165 researchers e STAMPEDE trial. In addition, the standardization of data collection methods and data transparency in single trials could further improve trial quality and facilitate the rapid approval of research protocols. In summary, the above features render platform trials an innovative and effective approach for clinical trials in precision medicine.

## Development: excavating therapeutic potential according to new biomarker-guided clinical trial design

The clinical popularization of NGS has revealed an abundance of potential therapeutic targets. However, it is unknown whether targeted therapy can achieve clinical benefits. The new clinical trial design guided by biomarkers efficiently explores the therapeutic potential of emerging gene-variation data. Based on the logic of drug-target relationships, tumor molecular typing, and dynamic changes, this paper summarizes the development achievements of basket, umbrella, and platform clinical trials.

### Basket trials unearthing the pan-cancer therapy of existing drugs based on the drug-target relationship

Based on the drug-target relationship, basket theory provides “targets search drugs” and “drugs identify targets” as two methodology models, extending drug indications for confirmed proliferation-driven targets and potential targets for proven effective drugs across different tumor species.

#### Targets search drugs

**Fig. 3:**
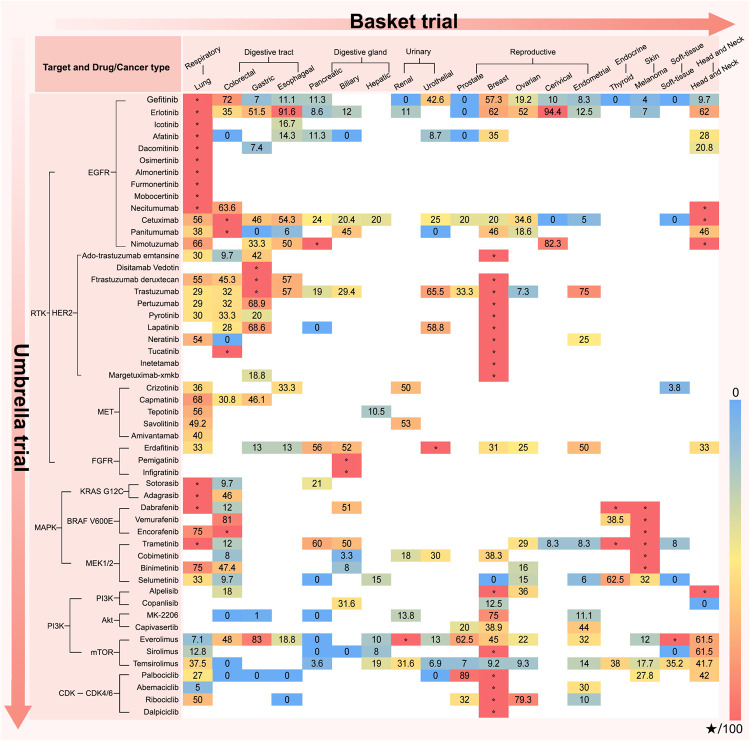
The efficacy and approval status of common targeted drugs in different tumor species. The number represents the ORR of drugs in different tumor species, with red indicating high response rates and blue indicating low response rates. The pentagram represents that the drug indication in a certain tumor species has been approved by FDA

The “targets search drugs” mode of basket theory focuses on targets with confirmed pan-cancer independent proliferation-driven ability, such as receptor tyrosine kinase families (e.g., EGFR, HER2, MET, and FGFR) and their downstream MAPK/PI3K pathway trunk signals (KRAS, BRAF, MEK, PI3K, AKT, and mTOR), and CDK4/6. Therefore, as long as potentially beneficial cancer species are identified, researchers can directly search for the corresponding drugs after detecting the targets (Fig. 3).

The “targets search single-drug” mode is undoubtedly the most classic pan-cancer treatment of the basket trial. By visually demonstrating the response rate and potential beneficial cancer species of common targeted drugs, we aim to assist cancer-related researchers in conducting large-scale clinical verification or new basket trials.

EGFR: EGFR is the most extensive proliferation-driven target in epithelial cancers, and its driving value extends from the first-line therapy for lung, colorectal, head and neck, and pancreatic cancers to cancers with squamous characteristics (head and neck carcinoma, cervical cancer, and Chinese esophageal cancer) and female reproductive system cancers (ovarian and breast cancers). Combined chemotherapy with EGFR-TKI prolongs the progression-free survival (PFS) of patients with NSCLC to almost 3 years.^116,117^ In addition, EGFR monoclonal antibody combined with chemotherapy has been approved as a first-line treatment for advanced colorectal cancer with KRAS wild-type, pancreatic, and head and neck cancers. Furthermore, in tumors with squamous characteristics, such as head and neck carcinoma, cervical, and Chinese esophageal cancers, the ORR of EGFR inhibition therapy combined with radiotherapy and chemotherapy is up to ~90%, showing improved survival compared to radiotherapy and chemotherapy alone.^118–121^ Similarly, the ORR of EGFR inhibition combined with chemotherapy for female reproductive system tumors, such as ovarian and breast cancers, also exceeds 50%.^122,123^

HER2: As the second-ranked molecule in the EGF receptor family, HER2 is recognized as a proliferation-driven target with overexpression (IHC2 + /3 + ) across multiple cancer types. Supporting the survival benefits for patients with breast and gastric cancers, anti-HER2 therapy has also achieved gratifying remission rates for lung,^124–127^ digestive tract tumors (esophageal, gastric, and colorectal cancers),^128–135^ digestive gland tumors (gallbladder and pancreatic cancers),^136,137^ and genitourinary tumors (breast, prostate, urothelial, and endometrial cancers).^138–141^

MET: MET, belonging to the HGF receptor family, was originally found in the form of a MET exon 14 skipping mutation in chemotherapy-insensitive tumors, such as liver cancer and renal cell carcinoma with poor prognosis, which could be reversed by MET inhibition therapy.^142–144^ Considering the transformative potential of MET in epithelial cells, MET inhibition has been validated in epithelial-derived tumors such as lung cancer and digestive tract tumors (gastric and colorectal cancers).^145–151^

FGFR: In addition to the evolution of tumor cells, fibrotic matrix remodeling mediated by FGFR activation leads to resistance. FGFR inhibition has been approved for FGFR2 amplification or mutation in urinary tumor and cholangiocarcinoma.^152,153^ Therefore, potentially beneficial tumor types may be those anatomical sites with a high degree of fibrosis caused by long-term chronic inflammatory stimulation, including pancreatic, lung, endometrial, and breast cancers, as well as head and neck squamous cell carcinoma.^153^

MAPK: The MAPK pathway, represented by RAS/RAF/MEK, has high evolutionary conservation and performs generalized functions, such as cell growth, differentiation, apoptosis, and migration by virtue of the widely expressed Ser/Thr kinase, which is the foundation of pan-cancer therapy. MAPK is the main downstream and compensatory activation pathway for ERGR, HER2, MET, and FGFR. Therefore, the tumor types that are likely to see the greatest benefit from KRAS, BRAF, and MEK-targeted therapy are similar to those of RTK families, mainly concentrated in lung and digestive system tumors.^83,154–162^ BRAF and MEK have also been approved as characteristic signals for BRAF-mutated solid tumors.

PI3K: PI3K/Akt/mTOR is another pan-cancer proliferation-driven pathway, and the downstream hub molecule mTOR has been functionally validated in most solid tumors. In addition, PI3K and Akt, which are characteristic resistance signals of breast cancer, are expanding the therapeutic potential of breast cancer to reproductive system tumors.^163–170^

CDK4/6: CDK4/6 inhibitor reverses the dilemma of endocrine resistance in breast cancer by alternating activation of CyclinD1 and ER.^171,172^ The CCND1 encoding CyclinD1 is a target gene of ER, and CyclinD1 can bind to ER to promote downstream gene expression of ER through a non-CDK-dependent pathway.^173^ According to interactions with hormone dependent pathways, CDK4/6 inhibitors are widely proven effective in reproductive system tumors.^174–180^

With the prolongation of survival and evolution of tumors under therapeutic stress, the proliferative signal network is intricate and ever-changing. Therefore, the low signal-blocking intensity and breadth of monotherapy will undoubtedly lead to a poor response or resistance.

According to multidrug synergy, the “targets search multi-drug” of basket trial is proposed to increase efficacy and delay drug resistance. On account of the inhibition of universal driving targets such as PD-1, MEK, and EGFR, the combined drugs design simultaneously cover tumor characteristics to ensure powerful and comprehensive signal blocking, achieving “high efficacy and slow resistance” across different tumor species.

PD-1 inhibitor-based therapy: PD-1 inhibitors improve the feasibility of the combination regimen for the universal mechanism among different tumor types. The phosphorylated intracellular structure of PD-1 mediates the dephosphorylation of downstream protein kinases Syk and PI3K, and further inhibits the activation of the AKT and ERK pathways, which can downregulate the expression of T-cell activation genes.^181,182^

The “omnipotent combination” of PD-1 inhibitors and multitarget TKIs is representative of the pan-cancer combined drug design, which shifts from posterior- to first-line stemming from their broad-spectrum antitumor activity.^183^ Multitarget TKIs promote vascular normalization to increase immune cell infiltration and improve the hypoxic microenvironment, thereby exerting synergistic efficiency enhancement. Moreover, multitarget TKI work quickly but are easily resistant, whereas PD-1 inhibitors take effect slowly but are unlikely to be resistant once effective. Therefore, this combination can cover the entire treatment process and achieve rapid and continuous remission. Specifically, apatinib inhibited PD-L1 expression in macrophages by targeting VEGFR2/STAT3 to reduce immune escape.^184–188^ Conversely, PD-L1 inhibitors can interact with VEGFR2 to block angiogenesis that is activated by the FAK/AKT pathway.^189,190^ Similarly, anlotinib reprograms the immunosuppressive microenvironment and increases immune cell infiltration to potentiate the therapeutic effect of PD-1 blockade.^191,192^ Apatinib combined with camrelizumab and anlotinib combined with cedilimumab have been validated in most solid tumors.^157,183,193–206^ Similar omnipotent combined drug design include lenvatinib with pembrolizumab and sulfatinib with toripalimab.

The PD-1, BRAF, and MEK inhibitor combined drug design blocks the synergistic enhancement of the oncogenic pathway and immune response in characteristic BRAF mutant tumors, including NSCLC, colorectal cancer, thyroid cancer, and melanoma. BRAF combined with MEK inhibitors has been approved for use in BRAF mutant solid tumors. BRAF upregulates PD-L1 expression by C-Jun via the MAPK/JNK pathway, which can be reversed by MEK inhibition.^167,207^ BRAF also upregulates PD-L1 expression through non-MAPK pathways, such as by activating IL-1 or LEF-1 transcription.^208,209^ Furthermore, BRAF responds to immunotherapy by inducing an IFN-γ-dominant immune microenvironment.^210^ Mutually, PD-1 inhibition reverses the exhaustion of CD8 + T cells induced by BRAF and MEK inhibitors.^167^ PD-1 inhibition suppresses the RAS/RAF/MAPK cascade by preventing SHP2 recruitment.^211,212^ The persistent immune response induced by PD-1 inhibition can also monitor reactivation of drug resistance pathways, including MAPK.^213^ Therefore, PD-1, BRAF, and MEK inhibitor combined therapies have been confirmed to be effective in BRAF-mutated colorectal cancer and melanoma.^211,214–216^

The PD-1 and KRAS inhibitor combined drug design also blocks the synergistic effect of the driving signal and immune escape in characteristic KRAS mutant tumors, including lung cancer and digestive tumors such as gastric cancer, colorectal cancer, and pancreatic cancer. KRAS upregulates PD-L1 expression by activating MEK/ERK, TGF-β/EMT, or YAP/TAZ transcriptional activators in lung cancer.^217–220^ Similarly, KRAS-driven pancreatic cancer is accompanied by PD-L1 overexpression caused by the deletion of the transcriptional suppressor TGIF1.^217^ The PD-1 and KRAS inhibitor basket has been demonstrated to be effective in KRAS mutant lung cancer and colorectal cancer.^221^

MEK inhibitor-based therapy: The MEK inhibitor-based therapy enhanced the blocking intensity of the MAPK pathway and predictably suppressed the alternative activation pathway to delay the occurrence of resistance, thereby improving the outcome of BRAF/KRAS mutant solid tumors that depend on the MAPK pathway.

The BRAF and MEK inhibitor combined design improves the efficacy of BRAF mutant tumors by blocking upstream and downstream signals. BRAF is a pan-cancer driver mutation with an incidence of 50% in melanoma and thyroid cancer and 10% in colorectal cancer.^29^ BRAF mutant tumors are MEK-dependent. Highly active mutants directly phosphorylate MEK through monomers or dimers, whereas low-activity mutants activate MEK through endogenous CRAF or RAS.^222–224^ Furthermore, resistance to BRAF inhibitors is also related to MAPK reactivation.^225–228^ However, the BRAF inhibitor vemurafenib or the MEK inhibitor trametinib alone failed in the cross-tumor exploration of BRAF V600E-mutated melanoma to other solid tumors.^229–232^ The failure of monotherapy suggests the necessity of increasing the blocking intensity of the MAPK pathway to accommodate differences across tumor species. Further trials have demonstrated that the combination of dabrafenib and trametinib, targeting both BRAF and downstream MEK, is effective in melanoma after BRAF inhibitor resistance.^233^ Similar combinations include vemurafenib with cobimetinib and encorafenib with binimetinib.^234,235^ The latter combination has also been validated in BRAF V600E mutant lung cancer and colorectal cancer.^158,236^ Moreover, the combined drug basket not only enhances the blocking intensity of the MAPK pathway but also magnifies its pro-apoptotic activity. BFAF inhibitors induce the ER stress response and upregulate the pro-apoptotic protein PUMA through the PERK pathway. MEK inhibitors can also mediate the expression of the pro-apoptotic protein BIM and the activation of the apoptosis mitochondrial pathway.^237^ As expected, the overall response rates of BRAF V600E solid tumors in the phase II ROAR (including cholangiocarcinoma, glioma, and thyroid cancer) basket trial and the NCI-MATCH sub-regimen are both around 80%.^83–86^ Subsequently, dabrafenib and trametinib baskets were approved in BRAF V600E mutant solid tumors, representing a defining change for the multidrug basket based on MEK inhibitors.

The EGFR, BRAF, and MEK inhibitor combined drug design blocks the downstream, upstream, and alternative activation signals to overcome the resistance of BRAF mutant tumors after combined treatment. BRAF mutant colorectal cancer has a high level of EGFR phosphorylation, which may induce the reactivation of ERK through EGFR-mediated RAS and CRAF activation to resist vemurafenib.^228,238^ However, a phase 2 basket trial of vemurafenib combined with cetuximab for treating BRAF V600E mutant non-melanoma cancer failed.^75^ Resistance may be attributed to MAPK reactivation and could not be reversed by adding chemotherapy.^157,239^ Furthermore, NRG1 derived from stromal cells can resist dabrafenib and trametinib through EGFR signal transduction.^238,240^ In other words, the combined inhibition of BRAF and EGFR leads to MEK-activated resistance, whereas combined inhibition of BRAF and MEK leads to EGFR-activated resistance. The failure of these clinical trials suggests the necessity of combined blocking of upstream, downstream, and alternative activation signals. Finally, encorafenib, binimetinib, and cetuximab basket were shown to prolong OS in patients with BRAF mutant colorectal cancer compared to standard treatment, which opens up new ideas for comprehensively blocking signal networks.^241^

The SHP2 and MEK inhibitor combined drug design has been shown to improve the efficacy of refractory KRAS-driven solid tumors by jointly blocking downstream and key node signals. SHP2 is not only the universal node of the MAPK pathway activated by different RTKs but is also the convergence node of many signaling pathways, including JAK/STAT, PI3K/AKT/mTOR, and PD-1/PD-L1. RAS-GTP-dependent carcinogenic KRAS mutation and amplification causes the full activation of upstream RTKs to converge to the RAS signal in pancreatic cancer, lung cancer, and melanoma cells.^242–247^ KRAS mutations also mediate acquired resistance to MEK inhibitors in NSCLC and thyroid cancers.^248–250^ SHP2 inhibitors can suppress the activation of KRAS mutants with GTP activity, and prevent SOS/RAS/MEK/ERK from responding to RTK reactivation induced by MEK inhibitors. The SHP2 inhibitor SHP099 combined with the MEK inhibitor trametinib has been shown to be effective in pancreatic, lung, and ovarian cancers with KRAS mutations and in triple-negative breast and gastric cancers with RAS amplification.^244,245^

EGFR inhibitor-based therapy: The EGFR inhibitor-based therapy blocks the same level of signal crosstalk to inhibit growth factor signal-dependent epithelial tumor proliferation. EGFR is the most significant proliferation-driven signal in epithelial tumors, with strong cross-tumor conservation. HER2, FGFR, MET, and VEGFR are the major alternative bypass signals that mediate resistance. Therefore, the foresighted combined inhibition of EGFR and accessory signals can improve the reduction rate and delay resistance of “dual signal addiction” tumors.

The EGFR and HER2 inhibitor combined drug design delays the resistance of EGFR/HER2-driven solid tumors due to the alternative activation signal within the ERBB family. ERBB2 mutations and amplification bypass activate downstream signals leading to EGFR inhibitor/antibody resistance to lung, colorectal, head and neck, and bladder cancers. HER2/HER3 dimerization activates the PI3K/AKT and ERK pathways to promote tumor growth in head and neck tumors after anti-EGFR therapy.^251,252^ HER2 D16 mutation promotes lung cancer resistance to osimertinib through an Src-independent pathway.^253^ HER2 L755S and R784G mutations are associated with anti-EGFR resistance in colorectal cancer.^254^ Bladder cancer cells resistant to cetuximab have high levels of ERBB2 phosphorylation and respond well to pan-HER inhibitors.^255^ Similarly, EGFR activation is the primary mechanism of anti-HER2 resistance in breast cancer and gastrointestinal tumors. ERBB mutations are present in 7% of HER2+ breast cancer cohorts and are resistant to HER2 inhibitors.^256^ Herceptin-resistant breast cancer cells show higher levels of EGFR phosphorylation and EGFR/HER2 heterodimers, with sensitivity to erlotinib and gefitinib.^257,258^ Overexpression of EGFR is observed in 26% of patients with HER2+ gastric cancer.^259^ Gastroesophageal cancer with co-amplified EGFR/HER2 is trastuzumab-resistant but sensitive to the pan-HER inhibitor afatinib.^260^ Lapatinib, may add therapeutic value in combination with trastuzumab through increased membrane HER2 levels and enhanced ADCC activity in breast cancer.^261,262^ Similarly, trastuzumab combined with lapatinib achieves regression of HER2-amplified gastrointestinal tumors by blocking HER3/EGFR reactivation.^263^ The activity of BIBW2292, AZD8931, AST1306, TAK-285, epertinib, and other EGFR/HER2 tyrosinase inhibitors in solid tumors indirectly confirms their interdependent proliferation-driven activity.^264–269^

The EGFR and FGFR inhibitor combined drug design prevents resistance from bypass activation in EGFR/FGFR dual-signal addictive tumors such as lung cancer, head and neck squamous cell carcinoma, hepatobiliary carcinoma, and esophageal cancer. The high expression/fusion of FGFR1 increases tumorigenicity and resistance to EGFR-TKI in EGFR mutant lung adenocarcinoma cells, which can be reversed by the dual inhibition of EGFR and FGFR.^270,271^ The combination of EGFR and FGFR inhibitors reverses EGFR resistance mediated by downstream PIK3CA activation in skin squamous cell carcinoma.^272^ Interactively, EGFR-dependent signal transduction induces resistance to FGFR inhibitors in head and neck squamous cell carcinoma cells with high expression of FGFR1 and cholangiocarcinoma cells with FGFR2 fusion.^273,274^ The inhibition of FGFR by lenvatinib results in the feedback activation of the EGFR–PAK2–ERK5 signal axis, which can be blocked by EGFR and EGFR inhibitors combined therapy.^275^ The combination of the FGFR inhibitor and gefitinib significantly decreased the levels of p-AKT and p-ERK1/2, inducing strong apoptosis and decreasing the ability of clone formation in esophageal squamous cell carcinoma.^276^

The EGFR and MET inhibitor combined drug design blocks MET co-activation through ligand-dependent or -independent pathways in EGFR-addicted tumors, including lung cancer, gastroesophageal cancer, colorectal cancer, and head and neck tumors. MET inhibition induces TGF-α to activate EGFR, resulting in bypass resistance in the abovementioned tumors.^277–279^ Conversely, EGFR inhibitors also induce HGF overexpression to bind to MET receptors that activate MAPK.^280^ Interestingly, TGF-α/HGF could activate MET/EGFR in both ligand-dependent and -independent manner.^281,282^ Therefore, inhibition of MET or EGFR alone is insufficient in MET/EGFR co-activated tumors. In MET amplification and EGFR mutation with T790M-negative NSCLC, the ORR of savolitinib combined with osimertinib is 64%,^283^ while the combination of tepotinib and gefitinib extends the OS to 24.2 months.^284^ Similarly, patients with NSCLC with EGFR mutant and high expression of MET treated with MET monoclonal antibody plus erlotinib exhibit a median PFS that is increased by 15.3 months compared to treatment with erlotinib alone.^285^ Moreover, combined treatment of MET inhibitor and cetuximab causes further tumor regression in patients with MET-positive colorectal cancer after anti-EGFR therapy.^147^ The bispecific antibody avantumab effectively downregulates the level of EGFR/MET activation and increases the immune directional antitumor activity induced by γ-IFN secretion.^286^ The ORR of avantumab reaches 40% in NSCLC with EGFR exon 20ins after platinum chemotherapy, and its potency in other tumors, such as gastroesophageal adenocarcinoma, is still being elucidated.^151,287^

The EGFR and VEGF inhibitor combined drug design blocks the energy loop enhanced by the cross-linking of epithelial proliferation and angiogenesis activation signals in EGFR-addicted tumors. Bevacizumab induces intracellular accumulation and activation of EGFR in colon cancer cells and tumor-associated endothelial cells, which can be attenuated by erlotinib, regardless of RAS status.^288^ Similarly, EGFR resistance in lung cancer may be related to increased VEGF expression in tumor and stromal cells.^289^ VEGF expression is regulated by EGFR signaling in a hypoxia-independent manner and remains high after resistance to EGFR inhibitors in EGFR-mutant lung cancers.^160^ The combination of bevacizumab and erlotinib basket has been validated in solid tumors, such as NSCLC, cholangiocarcinoma, liver cancer, breast cancer, head and neck carcinoma, glioblastoma, and anaplastic glioma.^290–294^ Other dural EGFR/VEGF inhibition therapy have also been proven effective, such as erlotinib plus cabozantinib in NSCLC, pazopanib plus cetuximab in head and neck squamous cell carcinoma, sorafenib plus cetuximab in colorectal cancer, pazopanib plus cetuximab in head and neck squamous cell carcinoma, and vandetanib in liver and thyroid cancer.^295–299^

The NCI and National Clinical Trials Network developed NCI-ComboMATCH in 2023, followed by a biomarker-guided study of NCI-MATCH, in order to address biomarker-guided drug synergies to increase efficacy.^300^ The therapeutic regime was supported by valid preclinical in vivo experimental evidence, consistent with the underlying logic of our proposed combined therapy. Further, ComboMATCH has both histology-specific and histology-agnostic arms, which reflects the comprehensiveness and inclusiveness of the new clinical design, including basket and umbrella trials. The publication of the ComboMATCH plan also manifested the recognition of the “targets search multidrug” model in basket trials by international institutions.

#### Drugs identify targets

The “drugs identify targets” mode of basket trials establishes therapeutic potential by identifying new targets of confirmed effective drugs across different tumor types. The detection of unknown gene variations by using NGS is the foundation for target expansion. Meanwhile, the pan-cancer application permission of the basket trial accelerates the verification of the therapeutic value of new targets. For example, PARP inhibitors expanded potential effective targets from BRCA2 to homologous recombination deficiency (HRD) and DNA repair-related genes.

From BRCA mutation to HRD and then to DNA damage repair-associated genes, PRAP inhibitors enlarge the pool of potential targets based on the generalizations of commonalities between individual cases. PARP can repair DNA single-strand breaks via the BER pathway. PARP inhibitors block BER, sequentially resulting in single-strand break accumulation, shortened replication forks, and formation of double-strand breaks. If BRCA mutations lead to HRD simultaneously, double-strand breaks mediated by PARP inhibitors will lead to cell death owing to their inability to be repaired. Olaparib and other PARP inhibitors have been approved for the treatment of advanced ovarian, breast, prostate, and pancreatic cancers with BRCA mutation.^301–307^ Unlike the “target search drug” mode, PARP inhibitors do not directly block the activation signal but anchor HRD to expand potential targets. Clinical studies have confirmed that PARP inhibitors offer clinical benefits to the above four cancers with other HRD-related genes (RAD51, ATM, and PABL2) and HRD without germline BRCA mutations.^308–315^ Clinicians have demonstrated that other DNA damage repair-related genes, such as SLFN11, indicate better efficacy of PARP inhibitors combined with temozolomide in small cell lung cancer (SCLC) and Ewing’s sarcoma.^316–318^

The basket trial has successfully opened up new opportunities for single-drug or multidrug therapies of rare tumors, which typically present challenges such as enrollment difficulties in traditional randomized trials. However, the trial type also has limitations. For example, replacing tumor tissue types with proliferation-driven molecular characteristics as treatment classification criteria is not always effective. Further, the gene-mutation spectrum of tumors is usually related to the site of tumor origin. Therefore, how to eliminate or master the negative impact of tumor tissue types on targeted therapy is a challenge and a major future research direction for basket trial design.

The internal logic of the success of the basket trial for tumors is that tumors have definite driving gene variations. In non-tumor diseases, if similar situations exist, patients may also benefit from a basket trial in the paradigm of “same drug for different diseases.” In neurodegenerative diseases such as Alzheimer’s disease, the biggest obstacle to performing a basket trial is the lack of sufficient biomarkers for most molecular pathologies besides Ab and tau. However, because both AD and certain non-AD neurodegenerative syndromes are strongly linked to underlying tau pathology, it is possible to combine populations such as AD, PSP, and corticobasal syndrome (CBS) in a single clinical trial of a tau-targeted intervention.^89^ Studies of drugs for infectious diseases or metastatic disease caused by the same pathogenic factor can also be done on the basket trial.^93^ In these studies, different stages of the disease were treated as different cohorts.^94^ When the concept of the basket trial extends to the research field of animal experiments, it can quickly help identify biomarkers.^92^

### Umbrella trial exploring tumor molecular-subtype-driven therapy

Umbrella trials allow the rapid validation of the effectiveness of multiple therapies or intervention for a certain disease, overcoming the limitation of traditional trial designs that only recruit patients who share common characteristics. These trials maximize the inclusion of individuals in the implementation of precision medicine, aiming to find the most suitable and personalized treatment strategies for each patient. Thus, it is essential for the application of an umbrella trial design to accurately and thoroughly identify the molecular biological feature of a specific disease, especially for precision-oncology research. Previously, numerous umbrella trials in the field of oncology have revealed many potential therapeutic strategies for cancer without a highly effective regimen.

There is currently no approved targeted therapy for squamous cell lung cancer. Despite The Cancer Genome Atlas (TCGA) project and other similar works detecting a high number of somatic gene mutations in squamous cell lung cancer, these molecular alternations occur at a relatively low frequency (5–20%), posing a significant challenge in rapid recruitment and efficient research if using traditional clinical trial design.^100^ Thus, the Lung-MAP (Lung Cancer Master Protocol) has emerged, which is a well-known umbrella trial for patients with squamous NSCLC that started in 2014.^100^ By incorporating multiple treatment options, investigators could simultaneously evaluate the biomarker-guided therapies more rapidly and without the need for separate trials for each regimen. This trial initially consisted of five arms, each investigating the efficacy and safety of a corresponding different approach. The first subgroup included patients without actionable molecular alterations of interest, who were assigned to receive durvalumab. Four additional subgroups were biomarker-driven and investigated targeted therapies including the PI3K inhibitor (taselisib) for PIK3CA alteration, CDK4/CDK6 inhibitor (palbociclib) for CDK4/CCND1/CCND2/CCND3 amplification, FGFR inhibitor (AZD4547) for EGFR1/2/3 alteration, and rilotumab and erlotinib for MET mutation (this arm was closed owing to toxicity).^319^ The Lung-MAP is a comprehensive umbrella trial that evaluates multiple targeted therapies and treatment approaches for squamous NSCLC patients. It utilizes biomarker-driven designs to match patients to specific interventions based on their molecular characteristics, ultimately improving treatment outcomes and advancing personalized medicine in lung cancer. Owing to the good design of Lung-MAP, this trial was expanded to include all of the histologic types of NSCLC in 2019 using a new screening protocol. The overarching aim of novel Lung-MAP is to evaluate multiple therapies and biomarkers in a single master protocol, facilitating personalized treatment approaches for patients with NSCLC.

Another important example is triple-negative breast cancer (TNBC). Although TNBC accounts for about 10–20% of all of the newly diagnosed breast cancers, patients with this type of breast cancer are prone to visceral metastasis. TNBC also comes with the highest risk of recurrence and the poorest survival rate of all breast cancers. Owing to lack of common breast cancer-associated targets such as the estrogen receptor, progesterone receptor, and HER2 expression, biomarker-guided treatment for patients with TNBC has been challenging. To address this issue, investigators have proposed the “Fudan Classification” based on multi-omics profiling of TNBC. In this classification system, patients with TNBC are categorized into four different subtypes: luminal androgen receptor (LAR), immunomodulatory (IM), basal-like immune-suppressed (BLIS), and mesenchymal-like (MES).^320^ Subsequently, the FUTURE trial using an umbrella design was initiated, in which previously heavily treated patients with TNBC were enrolled in four arms and received corresponding biomarker-guided therapy based on the FUDAN classification. The FUTURE trial found that the progression-free survival almost doubled compared with conventional chemotherapy.^320–322^ Therefore, the FUTURE trial has provided precision treatment options for patients with TNBC. This umbrella trial design has also offered a novel method for the efficient exploration of personalized treatment strategies.

The K-Umbrella trial for gastric cancer was carried out later, in which patients with gastric cancer were divided into three biomarker-guided therapy arms based on molecular subtyping (arm 1: EGFR IHC2+ or 3 + ; arm 2: PTEN loss/ineffectiveness; arm 3: immune-related biomarker enrichment including PD-L1 positive, microsatellite instability-high (MSI-H)/mismatch repair deficiency (dMMR), or EBV-related), as well as a control arm, with none of the abovementioned biomarkers.^323^ Similarly, the K-Umbrella trial aimed to simultaneously assess the efficacy improvement of three biomarker-based regimens. Even though this umbrella trial did not reach the study endpoint, it initiated the precedent of umbrella trials in a gastric cancer cohort. In addition, multiple umbrella trials are also being conducted to assess biomarker-guided therapy for ovarian cancer, urothelial cancer, and other diseases.^324,325^ The results of the above various umbrella trials allow us to realize the importance of accurate molecular profiling to achieve biomarker-based strategies in the era of precision medicine.

At present, tumor molecular profiling has become a focal point of research investment in line with precision medicine strategies. As the first comprehensive catalog of cancer-associated genomic alterations, TCGA has allowed researchers to explore genomic changes that may contribute to oncogenic phenotypes. The method uses genomic signatures to classify cancer at a molecular level, which has greatly enhanced the accuracy of selecting biomarker-guided therapies, thereby further improving the success rate of umbrella trials. Moreover, researchers can further establish tumor molecular profiling based on the vast amount of emerging omics data from advancements in next-generation sequencing and multi-omics technologies.

#### Lung cancer

The discovery of the common driver-gene therapeutic value, such as EGFR, ALK, and ROS1 in lung cancer, marked the initial exploration of umbrella trials based on molecular subtyping. The integration of comprehensive transcriptomic and epigenetic analysis, along with clinical-pathological data, has uncovered more intricate gene-network events and potential classifications. This advancement offers guidance for patients with lung cancer who do not harbor the defined driver genes. One novel classification has been proposed for this type of lung cancer, in which lung cancer is divided into three subtypes: Proximal-Proliferative (PP), characterized by KRAS mutations combined with STK11 inactivation; Proximal-Inflammatory (PI), characterized by NF1 and TP53 co-mutations; and Terminal Respiratory Unit (TRU), characterized by a high frequency of EGFR mutations.^326^ Similarly, the response to immunotherapy in KRAS mutant lung cancer is also closely linked to molecular subtyping. Different subtypes have shown significantly varying ORRs to PD-1 inhibitors: KL subtype (KRAS mutation with STK11/LKB1 co-mutations) had a rate of 7.4%, KP subtype (KRAS mutation with TP53 co-mutations) had a rate of 35.7%, and K-only (KRAS mutation alone) had a rate of 28.6%.^327^ All of these novel molecular subtypes have provided the possibility of conducting umbrella trials in common driver-gene-negative lung cancer, similarly to the FUTURE trial in TNBC.

#### Colorectal cancer

Colorectal cancer is another paradigm in the field of precision oncology. However, the previous biomarker-guided therapy for this cancer has been used for reverse selection of colorectal patients who are not responsive to targeted agents. For example, patients with colorectal cancer with the RAS/RAF-mutation could not obtain a clinical benefit from an anti-EGFR therapy should be treated with an anti-VEGF-targeted therapy.^328^ This molecular classification seems to be insufficient to support the application of a biomarker-guided therapy in the era of precision medicine. Thus, a more novel classification has been proposed. In 2015, the Colorectal Cancer Subtyping Consortium provided the clearest classification system for colorectal cancer to date.^329^ The consortium identified four molecular subtypes (CMS): the CMS1-MSI immune subtype, CMS2-Classic subtype, CMS3-Metabolic subtype, and CMS4-Mesenchymal subtype. Each subtype has distinct characteristics and potential benefits from specific biomarker-guided treatments. CMS1 has the characteristics of immune cell infiltration and the highest potential benefit from immunotherapy. CMS2 is primarily characterized by downstream targets of the WNT signaling pathway (APC gene) and high-frequency mutations in the p53 gene, which may benefit from treatments aimed at restoring the p53 function. CMS3 is the only subtype of the four with a high frequency of KRAS gene mutations, and RAS gene mutations have prognostic implications in colorectal cancer, predicting resistance to the anti-EGFR therapy in metastatic colorectal cancer. CMS4 is characterized by upregulation of EMT and stromal infiltration, which may be sensitive to therapy targeting WNT2. These classifications were developed in a preclinical setting, but further umbrella trials could explore biomarker-based therapy in accordance with molecular profiling.

#### Breast cancer

The most well-known molecular subtypes of breast cancer are Luminal A, Luminal B, HER2 overexpression, and TNBC, which have been widely used in clinical practice.^330^ In addition to these subtypes, as mentioned above, a novel molecular classification for TNBC, called “Fudan classification,” was developed based on clinical, genomic, and transcriptomic data.^320^ Despite hormone receptor-positive, HER2-negative breast cancer being the most prevalent form of breast cancer, the problem of resistance to endocrine therapy remains unresolved, highlighting the urgent need for accurate molecular classification to guide biomarker-based precision treatments. Based on the comprehensive omics data, investigators have classified HR + /HER2- breast cancer into four distinct subtypes: canonical luminal, immunogenic, proliferative, and receptor tyrosine kinase (RTK)-driven.^331^ Specific biomarker-guided treatment strategies would be developed based on the biological characteristics of each subtype. For example, the immunogenic subtype, which exhibits abundant immune cells, may produce a clinical benefit from an immune checkpoint inhibitor.

#### Gastric cancer

The failure of the K-Umbrella trial emphasizes the crucial importance of accurate and precise molecular classification for the successful implementation of an umbrella trial. Recently, multiple molecular-subtyping strategies for gastric cancer were proposed. TCGA proposed four subtypes of gastric cancer in 2014: genomic stability, chromosomal instability (CIN), microsatellite instability (MSI), and Epstein–Barr virus-positive.^332^ Unfortunately, the treatment-guided value of these molecular profiling types remains uncertain owing to the lack of adequate clinical data. In 2015, the Asian Cancer Research Group (ACRG) categorized gastric cancer into four subtypes, namely, MSI-H, MSS/TP53 + , MSS/TP53–, and MSS/EMT subtype, using gene-expression profiles, whole-genome-copy number-variation arrays, and targeted gene sequencing.^333^ The prognosis analysis demonstrated that patients with the MSI-H subtype exhibited the most favorable survival rate. In addition, gastric cancer has been classified into two groups using a predictive stratification based on genes related to immune function (GZMB and WARS) and intestinal epithelium (CDX1): one that would benefit from adjuvant chemotherapy and one that would not.^334^ Of course, this also requires the use of an umbrella trial design to effectively explore whether these novel preclinical molecular subtypes could guide clinical practice in the era of Precision Pro.

#### Biliary tract cancer

Biliary tract cancer is a highly heterogeneous malignant tumor at the genomic and epigenetic levels. With the development of gene-sequencing technology, multiple biomarkers that could guide targeted therapy or immunotherapy have been discovered, such as FGFR2 alteration, IDH1/2 mutation, NTRK fusion, RET fusion, BRAF V600E mutation, HER2 alteration, MSI-H/dMMR, and high tumor-mutation burden (TMB).^74,335–338^ As the traditional histopathological classification has exceeded the demands of precision medicine, a traditional “one-size-fits-all” trial also could not effectively assess the potential of a therapy target to the above molecular alterations. With the implementation of a new trial design for the umbrella trial, several biomarker-guided therapy approaches have been incorporated into clinical guidelines for cholangiocarcinoma. For instance, using the philosophy of an umbrella trial design, the FIGHT-202 trial aimed to explore the FGFR inhibitor in cholangiocarcinoma with FGFR gene alteration, including two genetic subpopulations: FGFR2 fusion or rearrangement and other FGF/FGFR alterations.^339^ In 2020 ASCO, an umbrella trial was designed to explore multiple biomarker-based therapy-target MET amplification, HER2 amplification, IDH1 mutation, and FGFR fusion among 46 patients with refractory biliary tract tumors, which yielded 26.1% of the ORR, with a median progression-free survival of 5 months.^340^ Except for these molecular events, there are several potential druggable genetic alterations in ongoing trials, such as EGFR, PI3K, and BRAF.^341^ Moreover, the molecular subtyping of cholangiocarcinoma has advanced into the field of multiple omics. For example, biliary tract cancer is now classified into five subtypes based on the tumor microenvironment, namely, immune classical, inflammatory stromal, hepatic stem-like, tumor classical, and desert-like.^342^ Each subtype has distinct characteristics, which can potentially guide trial design in a future umbrella trial.

#### Ovarian cancer

Biomarker-based therapy based on molecular heterogeneity has clearly emerged as a direction for precision treatment in ovarian cancer. Precision molecular subtyping forms the foundation for achieving individualized treatment for ovarian cancer. In 2011, TCGA categorized high-grade serous ovarian cancer into four subtypes based on gene content, namely, immunoreactive subtype, characterized by CXCL11/CXCL10/CXCR3 expression; proliferation subtype, characterized by HMGA2/SOX11/MCM2/PCNA overexpression with MUC1/MUC16 low-expression; differentiated subtype, characterized by MUC16/MUC1with SLP1-positive; and mesenchymal subtype, characterized by a HOX/stromal high-expression marker.^343^ The survival analysis showed that patients with the immunoreactive and mesenchymal subtypes had the best and worst prognosis, respectively. Further, Tan et al. reported a novel classification scheme to address the heterogeneity of epithelial ovarian cancer based on the gene-expression patterns of 1538 cases. Five subtypes, namely, epithelial-A, epithelial-B, mesenchymal, stem-like-A, and stem-like-B, exhibited biologically distinct characteristics and prognoses, as well as sensitivity to drugs.^344^ This molecular subtyping offers new insights into the development of biomarker-guided personalized therapy for ovarian cancer using the umbrella trial design.

#### Prostate cancer

Although precision medicine for prostate cancer started comparatively late, prostate cancer has also entered its era of precision medicine. The PROfound trial, released in 2020, is the first phase III clinical trial to explore biomarker-based therapy based on molecular subtyping in the field of prostate cancer.^303^ The PROfound trial has become a milestone in precision medicine for prostate cancer, leading to the approval of a PARP inhibitor (Olaparib) for advanced metastatic castration-resistant prostate cancer patients with BRCA mutations. The molecular subtyping of prostate cancer is mainly based on factors including transcriptomics, genomics, and proteomics. The molecular classification analysis based on this multi-omics information can help identify the gene-expression profile, activation status of oncogenes, DNA repair deficiencies, as well as protein expression, thus guiding the selection of biomarker-based therapies. For instance, BRCA1/2 gene mutations contribute to high sensitivity to PARP inhibitors.^345^ Moreover, PTEN gene loss is associated with the potential efficacy of PI3K/AKT/mTOR pathway inhibitors.^346^ Patients with the androgen-receptor variant may obtain a clinical benefit from androgen-receptor antagonists or CYP17 inhibitors.^346^ Similarly, therapy targeting the prostate-specific membrane antigen (PSMA), a prostate cancer-specific protein, is being tested in an ongoing trial. Owing to the high degree of genetic variation in metastatic castration-resistant prostate cancer, ~90% of patients harbor gene mutations with clinical significance.^347^ Therefore, using umbrella trial designs to evaluate the efficacy of multiple biomarker-guided therapies simultaneously is expected to become an effective approach for exploring precision treatment in prostate cancer.

#### Cervical cancer

The development of precision medicine in cervical cancer seems relatively slow, which can be attributed partly to the lack of an accurate understanding of the molecular subtype in cervical cancer. The accurate molecular classification can lay the foundation for therapeutic stratification of cervical cancer. Researchers have also made many attempts to explore the molecular subtyping of cervical cancer. For example, Li et al. reported a molecular stratification based on the data of single-cell transcriptomics and further identified four different molecular subtypes: hypoxia (S-H subtype), proliferation (S-P subtype), differentiation (S-D subtype), and immunoactive (S-I subtype).^348^ Moreover, patients with the S-H subtype, the S-I subtype exhibited the worst and best prognosis, respectively. In addition, different molecular subtypes presented various infiltrations of immune cells, especially for CD8 + T cells, suggesting immunotherapeutic potential of the immunoactive subtype. Currently, there is no standardized molecular subtyping for cervical cancer supported by relevant clinical trials. Cervical cancer exhibits a lot of genetic molecular alternations with unknown treatment values. The umbrella trials can help rapidly and effectively identify molecular alterations with significant treatment value.

In addition, molecular classification exploration is being conducted for many cancers, and an umbrella trial is an effective method for identifying a biomarker-guided therapy. Similarly, with the development of precise molecular-subtype exploration of the nononcology disease, the umbrella trial design would play a huge role in the precision treatment of these diseases.

### Platform trial screening of an optimal treatment in a long-term dynamic model

As a dynamic umbrella trial design, the platform trial is another new trial design that has revolutionized clinical research and drug development. The platform trial has broken from the traditional clinical trial model, using an adaptive clinical trial model designed to improve trial efficiency by minimizing the number of participants and shortening the time required to evaluate an experimental invention. Compared with umbrella trials, the standards for experimental intervention or controls are dynamically changing, resulting in a more effective way to screen out optimal treatment in a long-term dynamic model. Therefore, during the long process, the efficacy differences between different subgroups are dynamically quantified to ensure that the included patients receive the best treatment. The first platform trial, STAMPEDE, provides multiple standard treatment options for advanced prostate cancer.^108^ Subsequently, several platform trials have emerged in the field of precision oncology, although the number of this type of trial remains relatively small.^112,349^

The I-SPY 2 trial is widely regarded as another pioneer of tumor platform trials, which is designed to evaluate a neoadjuvant therapy in breast cancer, primarily to explore the effectiveness of different biomarkers and corresponding experimental drugs. First, a new patient with breast cancer is classified into one of 10 molecular subtypes.^349^ Then, I-SPY 2 trial’s adaptive randomization design enables this participant to be assigned randomly to a study arm. Initially, five experimental drugs can be efficiently, independently evaluated in parallel compared with the control, with a pathological complete response rate as the primary endpoint. During the course of the I-SPY 2 trial, the efficacy of each experimental drug is evaluated in a timely manner, and the study protocol can be adjusted based on the emerging evidence. For example, an experimental drug is considered successful if it achieves a predetermined level of effectiveness in one or more molecular subtypes. However, if it reaches a maximum number of participants without demonstrating any effectiveness, it may be terminated owing to futility. Throughout the trial, new experimental agents can enter into the trial by following a protocol amendment.

The I-SPY 2 trial provides several neoadjuvant regimens for different molecular subtypes of breast cancer in a single trial. In triple-negative breast cancer, the combination of veliparib-carboplatin and standard chemotherapy can result in significantly higher rates of a pathological complete response than standard therapy alone.^350^ Among patients with HER2-positive, HR-negative breast cancer, the I-SPY 2 trial revealed that neratinib added to standard chemotherapy was more likely to achieve a pathological complete response than standard chemotherapy with trastuzumab.^351^ In HER2-negative breast cancer, the combination of durvalumab and olaparib added to standard neoadjuvant chemotherapy showed superior efficacy to standard neoadjuvant chemotherapy, especially in a highly sensitive subset of patients with high-risk HR-positive/HER2-negative breast cancer.^352^ Moreover, MK-2206 (Akt inhibitor) combined with standard neoadjuvant therapy resulted in higher estimated rates of a complete pathological response in patients with HR-negative and HER2-positive breast cancer.^353^ The success of I-SPY 2 trial has highlighted the efficiency of umbrella trials in the field of precision medicine, specifically when exploring the use of molecular-biomarker-guided therapy.

The groundbreaking I-SPY 2 trial of neoadjuvant treatment for locally advanced breast cancer has established a new benchmark for the efficiency of phase II clinical trials. I-SPY 2 trial has several unique and novel aspects. First, it has an adaptive randomization method. The experimental drug group with a higher effective data receives more random patients. Second, it has a shared standard treatment control group. The shared standard treatment group avoids multiple enrollments of control group patients, improving the efficiency of the trial. Third, it employs the Bayesian decision method. If the experimental drug has a high Bayesian predictive probability of success in subsequent phase III clinical trials based on I-SPY 2, regimens will be moved from this trial and will enter the phase III trial. If the predictive success probability of the experimental regimen in phase III is low, this group will be stopped directly and withdrawn owing to futility. Fourth, the trial offers a dynamic adjustment of a trial protocol. According to the latest evidence, researchers have the capacity to promptly adjust the plan by increasing or reducing experimental regimens. I-SPY 2 trial continues to have a major influence on the development of next-generation trial designs in oncology and beyond.

Beyond oncology, platform trials are especially suitable for sudden public health emergencies that require effective treatment, like COVID-19, owing to the high efficiency of recruiting patients compared with conventional trials.^354–366^ In addition, platform trials can incorporate adaptive components whereby specific trial parameters may be altered during the course of the study if planned in advance. This kind of “rolling platform” can move on to test new drugs without stopping the trial or seamlessly including multiple stages of development.^367^ This is also good news for some diseases lacking effective treatment drugs, such as Alzheimer’s disease. For example, DIAN-TU is a platform trial that simultaneously evaluated solanezumab and gantenerumab in Alzheimer’s disease.^368^

## Direction: precision Pro, dynamic precision, and intelligent precision clinical trial design

In the past decade, since the master protocol-based novel clinical trial design was proposed, precision medicine has developed rapidly. New-drug efficacy verification has shifted from empiricism to biomarker-guided trials. Owing to the efficient and flexible design of the basket, umbrella, and platform trials, researchers have transformed generous drug targets into treatment opportunities. However, with the gradual exploration of simple targets and molecular typing, the dividend period of the current trial design methodology for research innovation has passed. The precision medicine era 1.0 of biomarker-guided new clinical trial design is also drawing to an end.

How to further thoroughly and precisely explore the therapeutic guidance value of bursting biomarkers is the most important proposition of clinical trial design in the era of precision medicine 2.0. The deep mining of genetic-variation data from multiple dimensions represents a necessary path for innovative new clinical design in the next decade. Moreover, to improve the therapeutic efficacy, both biological rationality and practical feasibility should be considered in the trial-design stage. Therefore, we propose that Precision Pro, Dynamic Precision, and Intelligent Precision be used to instruct new biomarker-guided clinical trial design in the precision medicine era 2.0.

### Precision Pro

Precision Pro refers to a treatment concept that explores optimal regimen closer to the essential biological mechanisms than current precision medicine. The Precision Pro concept re-evaluates characteristics of biological processes and involved molecules from multiple dimensions based on existing clinical and biological data. The Precision Pro concept will lead the second wave of new biomarker-guided clinical trials.

#### From a single target to multiple targets

**Fig. 4:**
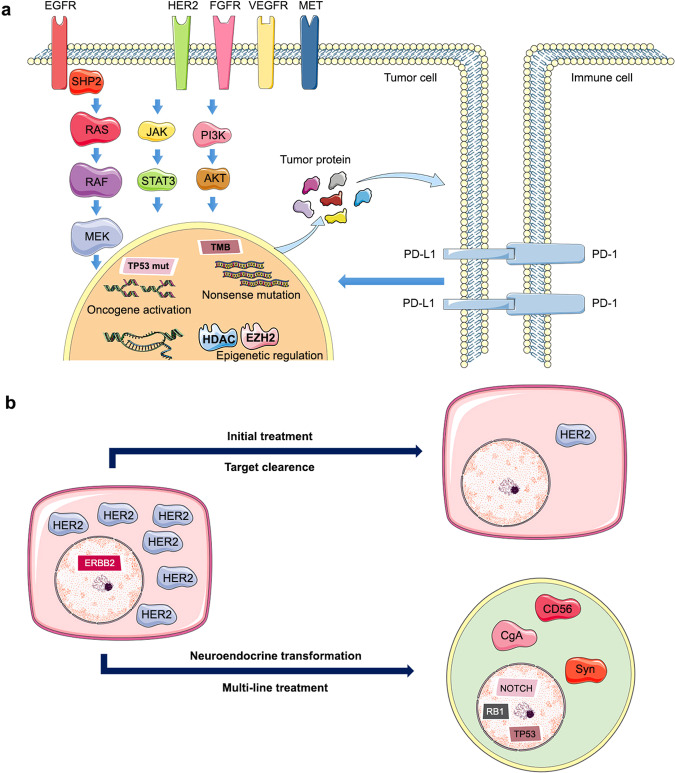
The molecular signaling pattern of the Precision Pro and Dynamic Precision. **a** The key signal transduction signaling with therapeutic potential from transmembrane to intracellular and nuclear. The combined application of TP53 and TMB accurately characterize the biological behavior of targets. The TP53 mutation and TMB serve as immunogenic backgrounds, presented in the form of extensive activation of oncogenes and nonsense mutations, respectively. The extensive genetic variations lead to the plenty production of tumor antigens and predict immunotherapy responses. The HDAC and EZH2 are the main epigenetic regulatory targets in nucleus with wide-ranging but relatively concentrated biological functions. The EGFR and HER2, FGFR, VEGR, MET are the main proliferation-driven transmembrane signals. The MAPK, JAK/STAT3, PI3K/AKT are the main intracellular proliferative signaling cascade pathways. The PD-1/PD-L1 is currently the most widely used immune checkpoint. **b** The signaling pattern transformation in Dynamic Precision. HER2 clearance occurs after initial anti-HER2 therapy, presented as protein inactivation and disappearance of original gene mutations. The genetic profiles and biologic characteristics of tumors change greatly after neuroendocrine transformation

Currently, most clinical trials focus on a single correspondence between the driving mutations and drugs. However, a comprehensive analysis found that genetic characteristics other than driving mutations were also related to curative efficacy and prognosis. Considering the impact of background gene features, including the TMB and TP53,^369,370^ on driving mutations, the combined targets improve the representativeness of the target to biological behavior and enhances its compatibility with differences in clinicopathological characteristics (Fig. 4a).

TMB combined with driver mutation: TMB, representing tumor immunogenicity, has been proven to predict the efficacy of immunotherapy for 27 tumor types.^79,371^ TMB, as a marker of new antigen and clone formation, can also assist in identifying tumor proliferation-advantageous clones.

Strong driver-mutated (such as EGFR, ALK, ROS1, MET, and RET) tumors have monoclonal growth advantages across tumor species, which suppress new mutations and clones, resulting in low TMB.^372,373^ The suppressive ability reflected by TMB positively correlated with single-drug basket therapy. Compared to those with high TMB, lung cancer patients with EGFR mutations and low TMB show a longer OS following treatment with EGFR-TKI.^126^ Similarly, patients with colorectal cancer with low TMB showed better efficacy when treated with EGFR monoclonal antibodies.^374,375^ Therefore, low TMB combined targets indicate that the signaling pathway activated by a strong driver mutation is the dominant factor in tumor proliferation.

Vulnerable driver-mutated tumors (e.g., BRAF, HER2, KRAS, and PI3KCA) require more mutations to gain survival advantages. The high TMB produced by nonsense mutations may dominate proliferation, exceeding that of driver mutations, indicating the benefits of immunotherapy.^372^ A high TMB has been shown to be associated with a low clinical benefit of EGFR/BRAF blocking therapy in patients with advanced colorectal with BRAF mutation.^375^ Moreover, lung cancer and melanoma with high TMB and BRAF mutations respond better to immune checkpoint blockade (ICB) therapy.^376–378^ Similarly, high TMB is an independent prognostic factor for KRAS mutant lung cancer and colorectal cancer treated with ICB.^376,379–381^ Furthermore, HER2 mutations are often associated with higher TMB in lung, breast, gastric, and colorectal cancers, all of which could benefit from ICB therapy.^382–387^ Anti-HER2 combined with PD-1 inhibitors has synergistic antitumor activity in both first- and posterior-line treatment of HER2-positive gastroesophageal adenocarcinoma.^388,389^ Therefore, although polyclonal proliferation driven by a high TMB responds poorly to single drugs, a higher level of antigen presentation tends to benefit from immunotherapy.

In summary, TMB condenses complex background genetic features into a single number, which is in accordance with the underlying logic of master protocol-based clinical trials. As a routine detection project for NGS, TMB has enormous potential for combined target applications.

TP53 combined with driver mutation: TP53 is a pan-cancer unfavorable prognostic gene that is mutated in 48.3% of solid tumors.^390^ TP53 and RAS mutations usually coexist in NSCLC, colorectal cancer, gastric cancer, pancreatic cancer, cholangiocarcinoma, and ampullary cancer, leading to increased invasiveness and shorter OS.^391–401^ However, TP53 wild-type can weaken the negative effect of RAS mutations on OS in lung and colorectal cancers.^391,397^ Accordingly, TP53 deletion may be more dominant than the driving mutation; thus, ignoring its combined predictive value may lead to a poor response.

TP53 combined with driver mutations exposes the weaknesses of strong tendency mutations, which can be exploited in immunotherapy. Although the extensive oncogenicity of TP53 and KRAS co-mutations enhances the proliferation and invasiveness of lung cancer and digestive system tumors, it also leads to higher immunogenicity and makes it easier to be recognized by immune cells. TP53 mutant lung cancer loses the binding constraint of BTG2 to RAS (G12V), resulting in a substantial increase in RAS proliferative activity.^402,403^ KRAS/TP53 co-mutation activates the Wnt/β-catenin pathway, leading to the separation of E-cadherin and β-catenin, thereby losing cell adhesion, and promoting invasion and metastasis in pancreatic ductal adenocarcinoma and gastric cancer.^404–406^ Similarly, TP53 deletion facilitates RhoA–ROCK pathway-dependent cell invasion and lymphatic metastasis in KRAS-mutated colorectal cancer.^407,408^ Fortunately, the widely oncogene expression activated by TP53/KRAS co-mutation also leads to the upregulation of immunogenic markers, such as TMB, PD-1, and MHC-I, and immunomodulatory factors, such as type I and type II interferons, indicating the potential benefits of immunotherapy.^409^ Clinical research has demonstrated that PD-L1 is upregulated in KRAS/TP53 co-mutated lung cancer and gastroesophageal adenocarcinoma,^410,411^ leading to better survival benefits with ICB therapy.

The discovery of the predictive value of high-frequency TP53 deletion in combined targets not only expands the potential beneficiaries but also avoids the poor efficacy of single-driver target suppression. Meanwhile, aiming functional enriched targets, such as TP53 and KRAS, further magnifies their proliferative advantages and reveals the weaknesses of immunogenic exposures behind their strengths. According to reverse thinking, the combined targets providing a unique approach for clinical trials to solve the bottleneck problem.

In clinical trial design, researchers should fully evaluate the sequencing data results and the biological implications of the enrolled patients. The accurate interpretation of biological-related data beyond therapeutic targets assists in precise treatment decision-making.

#### From the mutation to the mutation subtype

The salvageable structural p53 mutation subtype is screened by Arsenic trioxide from over 800 p53 mutations, which typically represents the “from the mutation to the mutation subtype” concept of Precision Pro. Although the mutation frequency of TP53 in advanced solid tumors is 48.3%, no drug targeting this mutation has been approved for 40 years.^390^ The failure of the eprenetapopt non-selective targeting of p53 mutation in the transition from phase II to III manifests the limitations of pan-target therapy.^412^ Essentially, the mechanism by which p53 mutations lead to functional loss varies, but includes unfoldable structural mutants, DNA-binding mutants with DNA-binding amino acid variation, and nonsense mutants.^413^ Arsenic trioxide, an approved therapeutic drug for acute promyelocytic leukemia, causes unfoldable p53 structural mutations to fold and restore its tumor inhibition function in the balance of denaturation and renaturation by releasing arsenic atoms and covalently bonding DNA-binding sites and β-sandwich domain in p53.^413,414^ Based on the logic of “from mutation itself to mutation subtype”, researchers have evaluated more than 800 TP53 mutants and found that 390 of them could be rescued by arsenic trioxide, of which 33 restored activities similar to the wild-type.^414^ The “PANDA” (P53 AND As) pan-cancer basket trial is currently being conducted to further verify the therapeutic value of arsenic trioxide in solid tumors with TP53 structural mutations. Similarly, the activator PC14586 selectively binds to the gaps in TP53 Y220C mutations to regain transcriptional activity, attaining an ORR of 24.2% in solid tumors.^415^ From the failure of general pan-targeting to the success of specific targeting of p53 mutates, the discovery of unknown structural variation through the “from mutation itself to mutation subtype” mode clarifies the beneficiaries of p53 inhibitors.

Similarly, the therapeutic potential exploration of pemigatinib among patients with cholangiocarcinoma in FIGHT-202 trial demonstrated the logic rationality of “*From the mutation to the mutation subtype*”.^339^ The different responses of FGFR subtypes to pemigatinib suggested varying drug responses in different molecular subtypes, even within the same gene alteration.

Therefore, the determination of mutation subtypes among enrolled patients should be based on understanding the biological nature of the different mutation subtypes of the targeted gene. Overly strict screening can lead to slow enrollment efficiency, while excessively broad filtering can reduce the overall effectiveness of the treatment.

#### From transmembrane signaling to nuclear signaling

In the era of precision medicine 1.0, new biomarker-guided clinical trials widely block transmembrane signals and downstream intracellular cascade reaction signals. From the perspective of stress selection, there is less intervention in intranuclear signaling, which maintains a relatively high therapeutic sensitivity. Moreover, nuclear signals, represented by epigenetic targets, are the starting point of the central rule, determining the biological phenotype executed by the vast majority of functional proteins. Research has found that the tumor microenvironment is also closely related to epigenetics. Moreover, epigenetic antitumor drugs have a wide range of immune regulatory effects. In summary, exploring the efficacy of nuclear signal regulation is an important direction for new clinical trial design guided by the Precision Pro concept.

The epigenetic drug therapy regulates the expression of multiple genes in nucleus to manipulate the tumor phenotype by targeting an HDAC or EZH2 gene-expression switch, initiating exploration of nuclear signal targeting (Fig. 4a). The complex drug resistance in the posterior line requires high-intensity combination rather than simple specific targeted therapy. HDAC inhibitors neutralize the positive charge of histone lysine by inhibiting its deacetylation, reducing its electrostatic attraction to negatively charged DNA, removing the entanglement between DNA and histones, and finally regulating the expression of multiple genes.^416^ EZH2 inhibitors relieve transcriptional inhibition of multiple genes by blocking PRC2 deposition mediated by the EZH2-mediated H3K27me3 complex.^417^ HDAC and EZH2 inhibitors can form the complex posterior-line state of tumors by reversing drug resistance, immune activation, and biological process enhancement through multi-gene regulation.

HDAC/EZH2 inhibitors reverse drug resistance by regulating the expression of multiple drug resistance-related pathway genes. HDAC inhibitors reverse endocrine drug resistance in breast cancer by reactivating ERα and aromatase expression.^418^ HDAC inhibitors also attenuate bypass-activated resistance to EGFR or MEK inhibitors by reducing the nuclear output of redundant tyrosine kinases in head and neck and colorectal cancers.^419,420^ Similarly, EZH2 inhibitors reverse chemoresistance by modulating local chromatin condensation and gene silencing, mediated by the downregulation of SLFN11 in SCLC.^421^ EZH2 downregulates MEIS1 transcription to maintain the integrity of DNA damage repair function, resulting in resistance to oxaliplatin in colorectal cancer, which can be reversed by EZH2 inhibitors.^254,422^ EZH2 inhibitors also reverse platinum resistance by downregulating the EZH2-mediated tumor suppressor DAB2IP in ovarian cancer.^423^ EZH2 induces tamoxifen resistance in breast cancer cells by silencing the expression of the ERα cofactor GREB1.^424^ EZH2 also inhibits RTK phosphorylation and overcomes alternatively activated sunitinib resistance in renal clear-cell carcinoma.^425^ Moreover, EZH2 downregulates the regulatory subunit of PP2A, PPP2R2B, resulting in sustained phosphorylation of the PP2A targets p70S6K and 4EBP1 to mediate anti-HER2 drug resistance in breast cancer.^426^

HDAC/EZH2 inhibitors enhance the efficacy of immunotherapy by upregulating the expression of inflammatory factors and immune-activating genes to reshape the proinflammatory tumor microenvironment. HDAC inhibitors stimulate the antitumor immune response by increasing the expression of proinflammatory chemokines and immunogenic cell death, thereby inducing proinflammatory tumor microenvironment in solid tumors such as triple-negative breast cancer, colorectal cancer, and adrenocortical carcinoma.^427–430^ EZH2 inhibitors decrease histone H3K27me3 modification on the β2-microglobulin promoter, leading to upregulation of MHC-I expression in head and neck squamous cell carcinoma and upregulation of MHC-II expression and immune cell infiltration in urothelial carcinoma.^431,432^ EZH2 inhibitors upregulate the NK cell-related genes MIP-1α, ICAM1, ICAM2, and CD86; activate NK cells in muscle-infiltrating bladder cancer; and upregulate the transcriptional level of the NKG2D ligand, which enhances the eradication of hepatoma cells by NK cells.^433,434^

HDAC inhibitors solve the therapeutic problems of undrugable targets by inducing cell death. HDAC inhibitors initiate the process of gene transcription of oxidative stress and apoptosis to treat undrugable targets, such as MYC, RAS, and NF1, in glioma, breast cancer, non-small cell lung cancer, and thyroid cancer.^435–438^

EZH2 inhibitors inhibit invasion-related pathways to reduce distant metastasis and improve disease control rates. EZH2 triggers SMAD3 methylation to promote the interaction between SMAD3 and its cell membrane locator, maintaining SMAD3 phosphorylation of TGFβ receptors and facilitating breast cancer metastasis.^439^ EZH2 also induces ribosomal synthesis overactivation and ribosomal DNA instability by silencing PHACTR2-AS1 to accelerate breast cancer metastasis.^440^ EZH2 induces methylation of lysine K362 in ERG, which is beneficial for DNA binding and increases ERG transcriptional activity, thereby enhancing the invasiveness of ERG fusion prostate cancer.^441,442^ EZH2 silences primary cilia genes and activates the Wnt pathway to promote melanoma metastasis.^443^

Clinical studies have confirmed that the EZH2 inhibitor tazemetostat has a good disease control rate in patients with epithelioid sarcoma and malignant pleural mesothelioma.^444,445^ HDAC inhibitors have also been shown to be effective in solid tumors, such as hormone-resistant melanoma, prostate, endometrial, and breast cancers.^446–449^ New clinical trials are expected to accelerate the clinical applications of epigenetic drugs. However, owing to the widespread impact of nuclear signaling on biological functions, serious side effects may occur. Therefore, in clinical trial design, a comprehensive evaluation should be conducted from the aspects of clinical drug accessibility, biological feasibility, and safety. Simultaneously, close monitoring of patients should be carried out.

#### From pan-cancer to relative specific cancer

As mentioned earlier, the “omnipotent combination” of PD-1 inhibitors and multitarget TKIs is representative of the pan-cancer drug combination. However, the “omnipotent combination” is not universally powerful for each cancer species. Clarifying the cancer relative specificity of combined therapy, especially the “omnipotent combination”, is an important mission assigned by precision oncology to new clinical trials design.

PD-1 expression specificity: PD-1 inhibitors, as a type of immunotherapy, stem from a universal biological mechanism and should be widely effective in pan-cancer therapies. However, the response rate to PD-1 inhibitors in advanced solid tumors is ~20%.^450–452^ Distinct PD-1 inhibitors were not significantly different because they share a similar mechanism. Successive studies have demonstrated that differences in efficacy may be attributed to varied immune characteristics represented by PD-1/PD-L1 expression across distinct anatomical tumor sites.

According to the distribution difference of PD-L1 on the surface of tumor cells and immune cells, the potential immune benefit tumors can be divided into tumor cell proportion score (TPS)-inclined and combined positive score (CPS)-inclined types. The TPS-inclined type includes NSCLC, melanoma, and renal cell carcinoma.^192,451,453,454^ Patients with positive tumor PD-L1 expression (TPS type) had a higher remission rate and were positively correlated with tumor quantity.^192,454–457^ However, further studies have shown that PD-1 is not only expressed in tumor cells, but also in tumor-infiltrating immune cells. The total level of PD-L1 expression in tumors and infiltrating immune cells (CPS type) is more sensitive than TPS in predicting the efficacy of PD-1.^453,458^ Clinical studies have confirmed the survival benefits of PD-1 inhibitors in CPS-inclined tumors, such as head and neck squamous cell carcinoma and esophageal, gastric, triple-negative breast, urothelial, cervical, and ovarian cancers.^191,459–466^

**Fig. 5:**
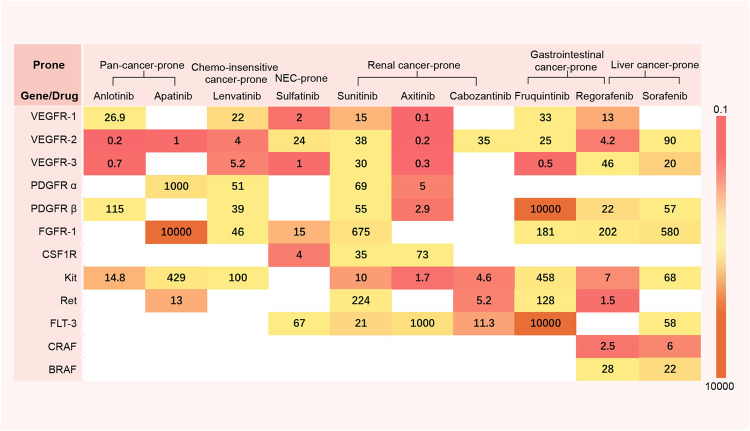
The inhibition power of common multitarget TKIs on different targets. The IC_50_ (nM) for different targets in different tumor types of multitarget TKIs are shown. The application preference of multitarget TKIs in multi-cancer with common characteristics has been summarized

Multi-target TKI specificity*:* Different cancer species show different responses in the clinical application of multitarget TKIs because of their different target spectra coverage. To optimize the selection of drugs in clinical practice, we divided multitarget TKIs in the “omnipotent combination” into six categories, namely, pan-cancer-prone (anlotinib and apatinib), neuroendocrine cancer-prone (sulfatinib), chemotherapy insensitivity prone (lenvatinib), renal cancer-prone (sunitinib, axitinib, and cabozantinib), gastrointestinal cancer-prone (fruquintinib and regorafinib), and liver cancer-prone (sorafenib and regorafenib), presenting with IC_50_ values for different targets (Fig. 5).^467–476^

The high-intensity blockade of VEGFR2 by the pan-cancer-prone TKIs, apatinib and anlotinib, empowers them with stronger cross-tumor differential compatibility. As mentioned for the combined drug basket, both anlotinib and apatinib can be used in the pan-cancer combined drug basket.

The chemotherapy-insensitive cancer-prone TKI lenvatinib tends to comprehensively inhibit various growth factors, including VEGFR/FGFR/PDGFR, which may dominate proliferation in chemotherapy-insensitive tumors.^477–479^ Lenvatinib combined with the PD-1 inhibitor pembrolizumab has been shown to be effective in metastatic liver cancer, renal cell carcinoma, melanoma, endometrial cancer, and gastric cancer.^480–484^

The neuroendocrine cancer-prone TKI sulfatinib monotherapy with a disease control rate of up to 90% in neuroendocrine tumors (NET) perfectly interprets its rationale for tumor specificity.^485^ Sulfatinib also reduces the progression risk of pancreatic and extrapancreatic NET by 51% and 67%, respectively.^486,487^ Furthermore, sulfatinib inhibits tumor invasion of myeloid-derived suppressor cells (MDSCs) by specifically targeting CSF-1R, reducing the number or changing the phenotype of tumor-associated macrophages, upregulating the ratio of CD8 + /CD4 + T cells, thereby improving the combined immunotherapy efficacy.^488–491^ The combination of sulfatinib and the PD-1 inhibitor treprinumab has an efficacy of 24% in advanced solid tumors (neuroendocrine cancer and gastrointestinal tumors with high neuroendocrine differentiation), in which standard treatment failed.^492^

Renal cancer-prone TKIs have clear therapeutic effects in renal cell carcinoma, and research on soft tissue sarcoma can draw on work related to renal cancer because of their similar pathological type. In addition to the inhibition of VEGFR2, sunitinib, axitinib, and cabozantinib also inhibit tumor proliferation by blocking the MEK/ERK and SAPK/JNK pathways and inhibiting the autophosphorylation of KIT, RET, and FLT3, which are usually activated in these two types of tumors.^493–496^ The efficacy of sunitinib, axitinib, and cabozantinib combined with PD-1 inhibitors has been confirmed in renal cell carcinoma and soft tissue sarcoma.^390,497–504^

Gastrointestinal cancer-prone TKIs, such as fruquintinib and regorafinib, combined with PD-1 inhibitors, have been proven to be effective in gastric and colorectal cancers.^505–508^ The therapeutic value of fruquintinib may have been underestimated in the past.

The liver cancer-prone TKIs Sorafenib and Regorafenib have been widely used to treat liver cancer. This clinical preference may be related to the rich blood supply to the liver, although the specific mechanism remains unclear. Sorafenib and PD-1 inhibition therapies improve the prognosis of patients with liver cancer.^509^ The survival benefit of sorafenib is positively correlated with the level of IFN-γ + /CD8 + T cells, whereas PD-1 inhibition enhances the local concentration of antineoplastic drugs by increasing blood perfusion through CD8 + T-cell accumulation and IFN-γ production.^510–512^ Similarly, regorafenib and PD-1 inhibitors are highly effective in the posterior-line treatment of liver cancer.^513^

The pan-cancer clinical design does not mean that tumor species should not be screened. Instead, the inclusion criteria for tumor species should be formulated based on a comprehensive consideration of the biological behavior of the tumor species and the characteristics of the drug. The cancer relative specificity constantly changes with ongoing preclinical and post-clinical evidence. Therefore, the cancer relative specificity concept is more important than the results we have summarized.

### Dynamic precision

Proliferation-dependent genetic characteristics of tumors constantly change dynamically under natural progression and therapeutic pressure. Accordingly, the drug-target relationship in new trial design should be considered with respect to variations in tumor characteristics. We focused on two types of dynamic target changes that have a considerable impact on the treatment strategy: from presence to absence, signifying the clearance of the driving target after treatment; and from absence to presence, signifying neuroendocrine transformation after treatment in non-neuroendocrine tumors (Fig. 4b).

#### Targets from presence to absence

HER2 clearance occurs in breast and gastric cancers with low target expression intensity but high anti-HER2 therapy intensity. After patients with HER2-positive early-stage breast cancer received T-DM1 or trastuzumab neoadjuvant therapy, the residual lesions in 8.3% of the patients became HER2-negative.^514^ HER2 also changed from positive to negative in 8.6% of patients with advanced breast cancer with bone metastasis.^515^ Similarly, in the T-ACT and GASTHER3 studies of advanced gastric cancer, 69% and 29.1% of patients treated with trastuzumab combined with chemotherapy became HER2-negative, respectively.^516,517^

However, the HER2-ADC still had strong disease control ability after the HER2 status changed from positive to negative. In patients with early breast cancer whose HER2 turned negative after neoadjuvant therapy, the 3-year disease-free survival of patients treated with T-DM1 was 100%, whereas that of patients treated with trastuzumab was only 70.1%.^514^ DS-8201 has also been approved for patients with breast cancer with low HER2 (IHC1 + ) expression based on DESTINY-Breast04 studies.^518^ Similarly, in the DESTINY-Gastirc01 study of advanced gastric cancer, the disease control rate of DS-8201 in HER2-negative (HER2 IHC ≤ 1 + ) patients was as high as 71.5%, and the median duration of response was 12.5 months.^519^

Target clearance after treatment is widespread in other tumor species and targets. For example, after first-line treatment of colon cancer, RAS and BRAF clearance occurred in 42.6% and 50% of patients with RAS and BRAF mutations, respectively.^520^ In addition, the incidence of microsatellite instability (MSI) in colorectal cancer was higher in the early stages than in late stages.^521^ Therefore, defining more characteristic tumor species and targets and further exploring treatment after target clearance is the future direction of dynamic precision clinical trial design. Furthermore, researchers should fully consider the potential impact of past treatments on the biological behavior of the tumor in a clinical trial design.

#### Targets from absence to presence

Neuroendocrine transformation characterized by presence of TP53 and RB1 variation is gradually becoming a common state of assimilation after multiline therapy. Profiting from the application of secondary biopsy after drug resistance, we observed the phenomenon of “neuroendocrine spectrum plasticity,” which means that non-neuroendocrine (non-NE) epithelial carcinoma changes to an invasive NE phenotype.^522^ Neuroendocrine transformation is widely found in solid tumors, such as lung cancer, prostate cancer, breast cancer, gastrointestinal tumors, and head and neck carcinoma, but it has poor efficacy owing to the lack of specific targets. Although other types of neuroendocrine carcinoma (NEC) can follow the treatment paradigm of small cell lung cancer (SCLC) according to the basket concept of the same treatment for different tumors, the efficacy of etoposide and platinum regimens in gastroesophageal, colorectal, pancreatic, prostate, large-cell lung, and other neuroendocrine cancers is limited.^523–526^

The entire gene-expression profile of tumors changes greatly after neuroendocrine transformation, along with the target feature changing from driving gene mutations to extensive gene variation.^527^ Moreover, mutations with complex functions, such as NOTCH inactivation and TP53 and RB1 deletions, have been found to play an important role in the transformation of SCLC and extrapulmonary small cell carcinoma.^528–531^ The extensive pattern of genetic variation in neuroendocrine cancer requires carpet-bombing therapy, based on immune checkpoint inhibitors combined with chemotherapy or anti-vascular targeting, to achieve long-term disease control. An etoposide and platinum regimen combined with ICB has been shown to achieve good efficacy and survival benefits in patients with advanced unresectable gastroenteropancreatic cancer, unidentified primary neuroendocrine neoplasms, and SCLC.^532–534^ The ORR of toripalimab combined with sulfatinib in patients with advanced NEC was 33%.^492^ Similarly, the ORR of atezolizumab combined with bevacizumab in pancreatic and extrapancreatic NET were 20% and 15%, respectively.^535^

The transformation of tumors into a state of multidrug resistance after multiline therapeutic pressure is the main evolutionary path of pan-cancer species. In addition to treatment pressure, tumor natural evolution may also result in changes in targets. For example, the HER2-positive rate in both advanced colon cancer and breast cancer were higher than that in early stage.^536–538^ Accordingly, identifying pan-cancer posterior-line homogeneity status and dealing with broad-spectrum biological effect drugs are meaningful directions for clinical trials in the era of precision oncology.

### Intelligent precision

Innovative thinking based on underlying biological logic is the primary version of new clinical design in the era of precision medicine 2.0. However, with the emergence of bioinformatics data brought about by technological progress, there is a strong demand for tools with powerful and flexible analytical capabilities. Further, complex and heterogeneous data in new clinical trials have increased this demand. Therefore, we propose the advanced version of “Intelligent Precision” to integrate intelligent technology support with a new clinical trial design.

Artificial intelligence (AI) and machine-learning algorithms have significantly improved the width and depth of data mining that traditional thinking models cannot accomplish. The disease diagnosis classification abilities of AI algorithms such as deep convolutional neural networks based on image recognition have been proven to be effective in the field of diabetes and oncology. The diagnostic accuracy is comparable to that of clinicians.^539,540^ The AI algorithms for disease progression modeling are also being developed to accurately characterize complexity and heterogeneity of neurological diseases, such as AD and Huntington’s disease.^541–543^ The formulation of more precise biomarkers and molecular subtypes in a clinical trial design will further improve therapeutic efficacy. Furthermore, intelligent technology also enables continuous data collection, reducing the negative impact of subjective measurements on data validity and regional barrier pressure on long-term research follow-up such as platform trials. Intelligent-precision-guided technology support can optimize the design of new clinical trials by analyzing and interpreting omics data and dynamic monitoring of patients, thereby assisting in the design of more feasible and effective trial protocols.

Intelligent precision incorporates histological heterogeneity in a basket trial design through comprehensive analysis of pan-cancer data. The pan-cancer data analysis of AI can reveal the heterogeneity of biomarkers between different diseases and guide the design of precise treatment plans. Researchers proposed the “comboSC” AI algorithm to 119 tumor samples of 15 tumor types, demonstrating its widespread practicality and superior performance in optimizing combination therapy plans in different cancer types.^544^

Intelligent precision identifies more accurate and diverse molecular subtype in an umbrella trial design through in-depth mining of multi-omics data. The analysis of genomic, transcriptome, and proteomic data by AI can fully reproduce the biological activity patterns from DNA to proteins. Researchers using spatial multi-omics techniques to analyze colon cancer samples have found that immune-exclusion (IEX) markers composed of DDR1, TGFBI, PAK4, and DPEP1 genes may become important predictors in the stratification of immunotherapy.^545^ In addition to bio-omics, clinical imaging omics data can also be fully utilized in an umbrella trial. The immune-consensus molecular subtype CMS (imCMS) of colon cancer based on AI algorithms in image-omics accurately classifies TCGA samples that cannot be classified by RNA expression profiles, compensating for the limitations of bio-omics in patient classification.^546^

Intelligent precision improves feasibility and patient compliance in long-term platform trial design through real-world data analysis. First, AI can efficiently estimate existing therapeutic efficacy data during platform trials (including successful and failed data) to verify the necessity of trial implementation. Second, in terms of patient recruitment, AI algorithms can search, analyze, and interpret big data such as electronic medical records, genetic testing omics data, and medical imaging data. Then, AI identifies potential clinical trial subjects and matches them with appropriate arms in platform trials, thereby significantly improving recruitment efficiency and reducing costs. Third, in terms of patient management, AI algorithms assist in continuously monitoring and managing patients through automated data collection in patient drug use, organ function, efficacy evaluation, adverse reactions, and other patient-centered data. Moreover, real-time and dynamic data monitoring further aligns with flexible designs represented by platform clinical trials, ensuring the accuracy of the verifying direction.

**Fig. 6:**
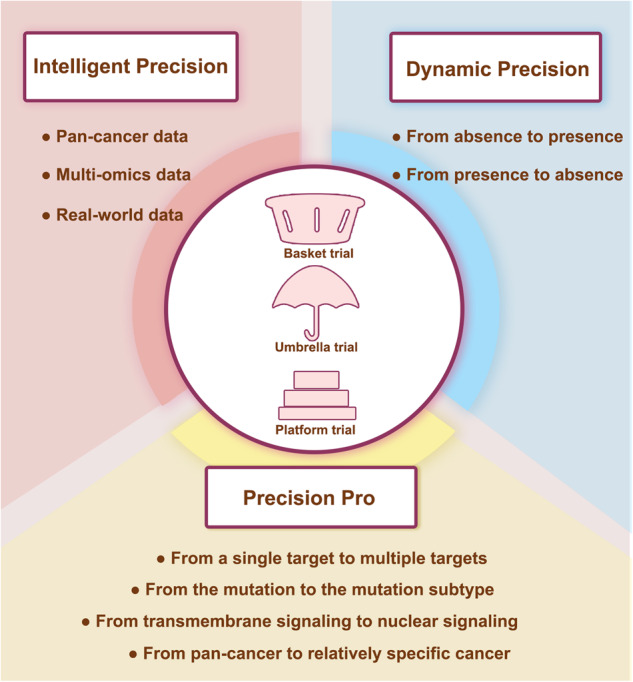
The future direction of new clinical trial design in precision medicine: Precision Pro, Dynamic Precision, and Intelligent Precision

Based on a full understanding of underlying biological mechanisms, the Precision Pro, Dynamic Precision, and Intelligent Precision concept will assist clinicians and trial-related researchers develop full-scale precise therapeutic strategies (Fig. 6).

## Conclusions and perspectives

In summary, the rapid development of high-throughput sequencing and multi-omics technology has created more possibilities for precision medicine. The new biomarker-guided clinical trial designs including basket, umbrella and platform trials have successfully transformed these possibilities into clinical benefits for patients. By sorting out the discovery and development of the new trial designs, trial-related researchers would be able to improve their understanding of this new methodology. However, with the gradual exploration of simple gene alterations and molecular typing, the innovation of trial methodology itself can no longer keep up with the individualized therapeutic demands. The cognition of the new biomarker-guided clinical trial design should not only focus on the primary idea of matching the targets, drugs, and diseases but also on the in-depth underlying biological logic of the origin and progression of diseases. The deep exploration of biomarker-related data will be full of opportunities for precision medicine in the next decade. We propose future direction for new clinical trials including Precision Pro, Dynamic Precision and Intelligent Precision. We look forward to jointly promoting clinical precision treatment from the perspectives of biological rationality and practical feasibility in the era of precision medicine.

The precise thinking model of biological mechanism-driven therapy will be the first principle in future clinical trial design. According to fully integrating theoretical innovation and intelligent technology to address the practical therapeutic demands of individual patients, the ability to control and manage disease precisely will be highly improved. The knowledge system construction based on new clinical trial design will assist researchers in grasping the decision occlusion and making a significant contribution to the rapid development of precision medicine era 2.0.
